# The conserved Tpk1 regulates non-homologous end joining double-strand break repair by phosphorylation of Nej1, a homolog of the human XLF

**DOI:** 10.1093/nar/gkab585

**Published:** 2021-07-09

**Authors:** Matthew Jessulat, Shahreen Amin, Mohsen Hooshyar, Ramy Malty, Mohamed Taha Moutaoufik, Mara Zilocchi, Zoe Istace, Sadhna Phanse, Hiroyuki Aoki, Katayoun Omidi, Daniel Burnside, Bahram Samanfar, Khaled A Aly, Ashkan Golshani, Mohan Babu

**Affiliations:** Department of Biochemistry, University of Regina, Regina, Saskatchewan S4S 0A2, Canada; Department of Biochemistry, University of Regina, Regina, Saskatchewan S4S 0A2, Canada; Department of Biology, Carleton University, Ottawa, Ontario K1S 5B6, Canada; Ottawa Institute of Systems Biology, University of Ottawa, Ottawa, Ontario K1S5 B6, Canada; Department of Biochemistry, University of Regina, Regina, Saskatchewan S4S 0A2, Canada; Department of Biochemistry, University of Regina, Regina, Saskatchewan S4S 0A2, Canada; Department of Biochemistry, University of Regina, Regina, Saskatchewan S4S 0A2, Canada; Department of Biochemistry, University of Regina, Regina, Saskatchewan S4S 0A2, Canada; Department of Biochemistry, University of Regina, Regina, Saskatchewan S4S 0A2, Canada; Department of Biochemistry, University of Regina, Regina, Saskatchewan S4S 0A2, Canada; Department of Biology, Carleton University, Ottawa, Ontario K1S 5B6, Canada; Ottawa Institute of Systems Biology, University of Ottawa, Ottawa, Ontario K1S5 B6, Canada; Department of Biology, Carleton University, Ottawa, Ontario K1S 5B6, Canada; Ottawa Institute of Systems Biology, University of Ottawa, Ottawa, Ontario K1S5 B6, Canada; Department of Biology, Carleton University, Ottawa, Ontario K1S 5B6, Canada; Ottawa Institute of Systems Biology, University of Ottawa, Ottawa, Ontario K1S5 B6, Canada; Department of Biochemistry, University of Regina, Regina, Saskatchewan S4S 0A2, Canada; Department of Biology, Carleton University, Ottawa, Ontario K1S 5B6, Canada; Ottawa Institute of Systems Biology, University of Ottawa, Ottawa, Ontario K1S5 B6, Canada; Department of Biochemistry, University of Regina, Regina, Saskatchewan S4S 0A2, Canada

## Abstract

The yeast cyclic AMP-dependent protein kinase A (PKA) is a ubiquitous serine–threonine kinase, encompassing three catalytic (Tpk1–3) and one regulatory (Bcy1) subunits. Evidence suggests PKA involvement in DNA damage checkpoint response, but how DNA repair pathways are regulated by PKA subunits remains inconclusive. Here, we report that deleting the *tpk1* catalytic subunit reduces non-homologous end joining (NHEJ) efficiency, whereas *tpk2-3* and *bcy1* deletion does not. Epistatic analyses revealed that *tpk1*, as well as the DNA damage checkpoint kinase (*dun1*) and NHEJ factor (*nej1*), co-function in the same pathway, and parallel to the NHEJ factor *yku80*. Chromatin immunoprecipitation and resection data suggest that *tpk1* deletion influences repair protein recruitments and DNA resection. Further, we show that Tpk1 phosphorylation of Nej1 at S298 (a Dun1 phosphosite) is indispensable for NHEJ repair and nuclear targeting of Nej1 and its binding partner Lif1. In mammalian cells, loss of *PRKACB* (human homolog of Tpk1) also reduced NHEJ efficiency, and similarly, PRKACB was found to phosphorylate XLF (a Nej1 human homolog) at S263, a corresponding residue of the yeast Nej1 S298. Together, our results uncover a new and conserved mechanism for Tpk1 and PRKACB in phosphorylating Nej1 (or XLF), which is critically required for NHEJ repair.

## Graphical Abstract

**Graphical Abstract:**
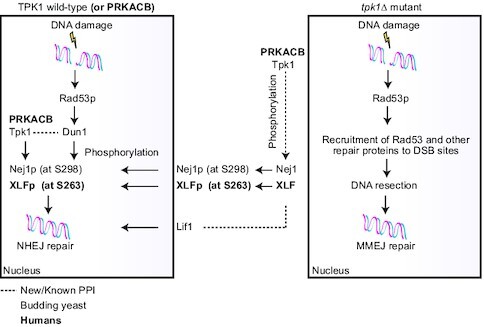
Cellular model of yeast Tpk1 role with Nej1 and PRKACBwith XLF on NHEJ, as well as the loss of tpk1 in DNA resection and MMEJ.

## INTRODUCTION

DNA double-stranded breaks (DSBs) are the most severe forms of DNA damage, causing genomic instability and chromosomal rearrangements (1), leading to serious human diseases including cancer and immunodeficiency response (2,3). In response to DSBs, eukaryotic cells recruit three repair pathways: homologous recombination (HR), non-homologous end joining (NHEJ), and microhomology-mediated end joining (MMEJ) to rescue genomic instability. NHEJ is the main DSB repair pathway in mammalian cells, and highly conserved in eukaryotes ranging from the budding yeast, *Saccharomyces cerevisiae*, to humans (4). In yeast, DSB repair through NHEJ requires three (Yku, MRX, DNA ligase IV) different macromolecular complexes for repair initiation, ligation preparation, and rejoining of broken ends, respectively.

Recognition of DNA damage involves recruitment of the multifunctional MRX (Mre11–Rad50–Xrs2) complex (5), the Yku DSB end-binding complex (Yku70–Yku80) (6), and a cascade of DNA damage checkpoints in which Mec1 and Rad53 play central roles (7). The MRX complex, in preparation for ligation, forms a bridge between the broken ends and brings them to close proximity (8). The final rejoining step requires the DNA ligase IV complex (Lif1–Lig4/Dnl4), in which a third yeast NHEJ protein, Nej1, interacts with Lif1 (9), thereby recruiting Pol4 and Rad27 proteins for NHEJ end processing and DSB repair (10). In addition, Nej1 mediates Yku retention at DSBs in a DNA ligase IV-dependent manner (11). In response to DNA damage, Nej1 is phosphorylated on S297/298 by the Dun1 checkpoint kinase (12), but additional contributors to Nej1 phosphorylation and NHEJ-mediated repair remain unknown.

The cyclic adenosine 3′, 5′-monophosphate (cAMP)-dependent protein kinase A (PKA) is one of the primary kinases required for several functions in the cell, including regulation of DNA repair (13,14). In yeast, PKA is composed of three catalytic subunits, encoded by *TPK1-3* genes, and one regulatory subunit, encoded by *BCY1* gene (15,16). Components of the PKA catalytic subunit trimeric complex (Tpk1-3) exhibit varied substrate specificities (17), indicating the potential for distinct functional profiles. A yeast proteome chip screening approach (17) has uncovered Nej1 as a phosphorylation substrate of Tpk1, but not Tpk2 or Tpk3, implying a likely role for Tpk1 in DNA repair. Our recent work has shown *tpk1* deletion impaired NHEJ (18), and that it is epistatic with a yeast uncharacterized gene, *hur1* (HU resistance 1) in NHEJ, though the molecular mechanism of this connection in DNA repair remains unclear (19). While this and other evidence suggests the role of yeast PKA in DNA damage checkpoint pathways (13), and Tpk1 in NHEJ (18,19), how PKA subunits impact these processes, and whether there is a conserved role between yeast and human in DSB repair pathway, warrants further investigation.

In the present study, we show that deletion of *tpk1* and *nej1* (or the checkpoint kinase, *dun1*), alone or in combination, reduces NHEJ efficiency by participating in the same pathway, while functioning redundantly to *yku*80. We further demonstrate that yeast Tpk1 exhibits an *in vitro* kinase activity dependent on Nej1 serine residue at S298, a phosphosite of Dun1, suggesting a crosstalk between the two DNA damage checkpoint kinases (Tpk1, Dun1) through shared phosphorylation of the Nej1 S298. As well, the repair defect of *tpk1* deletion can be recovered by mutation of Nej1 S298E (phosphomimetic), but not Nej1 S298A (non-phosphorylatable), consistent with the phosphorylation of S298 being a requisite for Tpk1’s role in NHEJ repair. As in yeast, PRKACB, a human homolog of Tpk1, showed similar NHEJ defect in DSB repair by phosphorylating the Nej1 human homolog, XLF at S263, suggesting a conserved NHEJ role for Tpk1 in eukaryotes. Overall, our findings suggest that Nej1 phosphorylation by Tpk1 is intrinsic to NHEJ break repair and resolution, and highlight a conserved model for NHEJ regulation.

## MATERIALS AND METHODS

Besides the procedures described below, site-directed mutagenesis, *in vitro* kinase and phosphorylation-induced mobility shift, sensitivity to DNA damage, clonogenic survival, cell proliferation, phosphoproteomics/affinity purification coupled with mass spectrometry (MS), immunoblotting, purification of yeast Tpk1 and Nej1 recombinant proteins, generation of stable CRISPR gene knockouts (KOs), immunofluorescence staining and quantification of DNA damage foci, and alkaline comet assay, among others are detailed in Supplementary Methods.

### Mutant strains or CRISPR gene KOs, cell cultures and plasmids

All yeast gene deletions (or mammalian gene KOs), plasmids, primers, and antibodies used are listed in Supplementary Table S1. Yeast FLAG or HA-tagged fusion proteins and mutant strains were created in BY4741 or JKM139 background by lithium acetate transformation (20). Deletions were confirmed by PCR and/or DNA sequencing across the deletion site. Yeast strains were grown in YPD (1% yeast extract, 2% bacto-peptone, 2% glucose) at 30°C, unless otherwise noted. Human osteosarcoma U2OS and HEK293T cells were cultured in Dulbecco's modified Eagle's medium supplemented with 10% fetal bovine serum and 1% penicillin-streptomycin mix at 37°C and 5% CO_2_. Stable human gene KOs were created using a CRISPR-Cas9 system with LentiCRISPR-v2 blasticidin or puromycin, following established procedure (21).

### NHEJ or HR assays and plasmid mutagenesis

The plasmid end joining or chromosomal DSB repair for NHEJ, plasmid mutagenesis, and HR plasmid repair assays in yeast were performed as previously described (20). Briefly, circular plasmids pRS416 and Ycplac111 with URA3 or LEU2 selective markers for relevant background strains were digested at their unique *Xba*I or *Sma*I restriction sites. About 20 ng of circular and linearized plasmids were transformed using lithium acetate, and selected on minimal media lacking uracil or leucine. Colony formation from the replicates of linear/circular transformations for mutant over linear/circular for wild-type managed on the same day were used to estimate NHEJ repair efficiency. For plasmid mutagenesis, XbaI or SmaI-digested pRS416 plasmid was transformed into the yeast wild-type or mutant strains, and plasmid DNA recovered from at least 24 single yeast positive colonies after URA3 selection were transformed into the chemically competent DH5α cells, following the manufacturer's instructions. Plasmid DNA isolated from successful transformants was purified using QIAprep spin miniprep kit (Qiagen), and analyzed after sequencing at Toronto's TCAG (The Centre for Applied Genomics) facility.

For chromosomal DSB repair, overnight cultures of the wild-type JKM139 strain, gene deletion mutants, and those carrying overexpression plasmids were grown in YPD at 30°C to an OD_600_ of ∼0.5. Harvested cells were washed in sterile water three times to remove residual glucose prior to transferring to non-repressing YPEG media (YP plus 2% ethanol and 3% glycerol), and grown to the logarithmic phase with constant shaking at 30°C. The cultures were serially diluted from 10^–1^ to 10^–6^ cells, and 100 μl of each dilution was plated on YP medium containing glucose or galactose to generate HO-induced DSBs. After incubating the plates for 2–4 days at 30°C, chromosomal DSB efficiency reflects the colonies survived in galactose over glucose media.

HR plasmid repair was carried out by co-transforming 10 ng of BglII-digested linear pGV256-DEAD plasmid DNA and 200 ng of the purified *lacZ* amplicon (PCR amplified from pGV255-LIVE plasmid using *lacZ* primers) into the yeast mutant strain using a standard transformation procedure, with at least 50 colonies assayed for *lacZ* reporter activity via colony-lift filter assay (20). HR efficiency was calculated as the number of *lacZ*-producing blue colonies over a circular plasmid transformation with uncut pGV256 plasmid, and normalizing mutants to wild-type.

### Chromatin immunoprecipitation (ChIP) and DNA resection

ChIP was performed as described (20) with the following modifications. A 5 ml overnight culture of Nej1-HA or Nej1-HA in *tpk1* deletion of JKM139 cells was grown in non-repressing YPEG media to the mid-logarithmic phase. To induce HO cleavage and overexpression of Nej1-HA construct carrying GAL1 promoter, the culture was spiked with 2% galactose for 1 h. Following 1% formaldehyde crosslinking for 30 min and reaction quenching with 150 mM glycine, cells after washing were disrupted with glass beads, and the extracts were instantly processed for sonication. The sonicated chromatin was immunoprecipitated with protein G beads (Miltenyi) and an anti-Tpk1, anti-HA or anti-Yku70 (Santa Cruz) antibodies.

DNA resection was assayed as previously described (22) in JKM139 strain grown in YPEG media to the logarithmic phase. DNA damage was induced with 2% galactose, and 50 ml was taken at each time point. Cells were resuspended in ice-cold lysis buffer (20 mM HEPES KOH pH 7.4, 150 mM KOAc, 2 mM MgAc2, 1 mM EGTA, 0.6 M sorbitol), followed by mechanical glass bead disruption and 5 min of vortexing. Chromosomal DNA from the lysate was isolated via phenol-chloroform extraction. Purified DNA was resuspended in 100 μl of water, and approximately 1 μl of DNA was used for restriction enzyme digestion using *Psi*I, which cuts once at each DNA locus if the DNA is in a double-stranded conformation, thereby preventing quantitative real-time PCR (qRT-PCR) amplification. Control (uncut) DNA was processed in the same way (2 h incubation at 37°C) but in the absence of the restriction enzyme. QRT-PCR was performed using a LightCycler96 (Roche) and the FastStart Essential DNA Green master mix (Roche) as per manufacturer's instructions. ChIP results are displayed as fold-enrichment at the target (i.e. DSB) site relative to a control locus, while percent linearized DNA for resection was calculated from the increase in Ct cycles compared to control uncut DNA.

### *In vivo* NHEJ and HR assays in mammalian cells

The NHEJ reporter plasmid (pEGFP-Pem1-Ad2) provided by V. Gorbunova (University of Rochester) as described (23) was digested at 37°C with I-*Sce*I homing enzyme. This linearized pEGFP-Pem1-Ad2 (green) was co-transfected with pDsRed2-N1 (red, Clontech) plasmid in 80–90% confluent U2OS cells (3 × 10^6^ cells in 60 mm plate) carrying control sgRNA or CRISPR gene KOs. In the case of HR reporter assay, control sgRNA or CRISPR gene KO U2OS cells were co-transfected with plasmids containing pCAGGS-mCherry as well as pDRGFP and pCBASceI, expressing I-SceI endonuclease from a mammalian promoter that introduces DSBs at genomic I-SceI sites (24). All plasmids were transfected in control sgRNA or gene KO U2OS cells using Lipofectamine^®^ LTX with PLUS transfection reagent (ThermoFisher Scientific), following the manufacturer's instructions. After 72 h of transfection, harvested cells were washed in phosphate-buffered saline and fixed in 2% paraformaldehyde for Fluorescence-activated cell sorting analysis using a MoFlo XDP Flow Cytometer (Beckman Coulter). The repair efficiency in wild-type or gene KOs was reported by measuring cells positive for both GFP and DsRed (or mCherry) over DsRed or mCherry positive cells.

## RESULTS

### Yeast Tpk1 is required for chromosomal DSB repair by NHEJ

**Figure 1.**
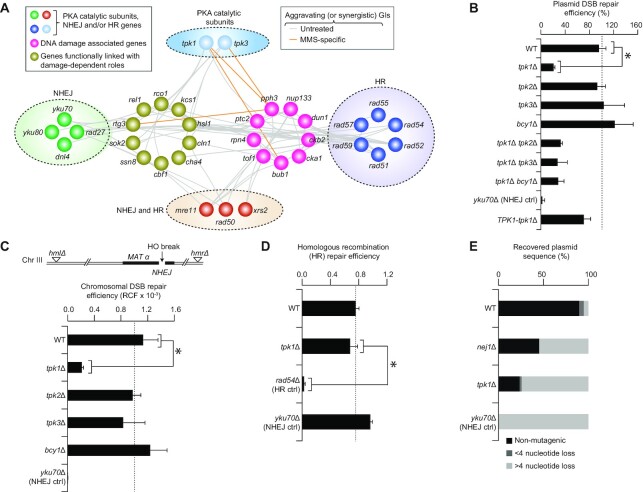
Tpk1, but not other PKA components, associates with NHEJ. (**A**) PKA catalytic subunits, HR, NHEJ, and MRX (i.e. both NHEJ and HR) genes sharing synergistic genetic interactions (GIs; see Supplementary Table S2) with DNA damage-related genes in untreated and specific to MMS treated conditions (25,26). (**B, C**) Repair efficiency for plasmids (B) and HO-induced chromosomal (C; RCF, relative colony formation) DSBs on wild-type (WT) and mutant strains. Schematic (top; panel C) of the yeast JKM139 strain on chromosome III, expressing galactose-inducible HO endonuclease at *MAT* locus, bearing *hml* and *hmr* deletions. (**D**) HR efficiency of the WT and mutant strains. (**E**) Plasmids recovered (*n* ≥ 20 clones) from WT and mutant strains. Data in panels B–D are mean ± SD (*n* = 5 biological replicates; *P ≤ 0.05 by Student's *t* test).

Since our recent work suggests that Tpk1 is a potential contributor to DSB repair (18), we examined genetic interactome investigations (25,26) under genotoxic stress or standard laboratory growth conditions, which revealed that tpk1 and *tpk*3, as well as components of the NHEJ (*dnl4*, *rad27*, *yku70/80*), HR (*rad51/52/54/55/57/59*) or NHEJ and HR (*mre11, rad50*, *xrs2*) DNA repair machineries shared 87 aggravating (i.e. combination of mutations impairing cell growth; referred as synergistic, Supplementary Table S2) genetic interactions, with 19 genes involved in DNA damage and other processes linked to damage-dependent roles (25,26). This includes the functional connectivity between *tpk1*/*tpk3* and *Dun1* or *yku70* to genes involved in DDR (DNA damage response; *rtg3*) and NHEJ (*pph3*; Figure 1A). These observations suggest that the PKA catalytic subunits are either involved in NHEJ repair or broader DNA DSB repair mechanisms.

To examine the possible role for all PKA components in mediating genome integrity, the *tpk1-3* deletion mutants were tested for efficient DSB repair using an established plasmid repair assay (20). Essentially, NHEJ is measured by transforming linearized and circularized plasmids into mutant or wild-type strains in parallel, and mutants exhibiting low tendency to form colonies under linear DNA transformations were considered to be defective in NHEJ. As before (18), strains lacking *tpk1* exhibited poor repair efficiency of linearized plasmids with four nucleotide (5′-GATC) overhang, albeit not to the same extent as with the *yku70*-deficient NHEJ mutant (Figure 1B). However, native expression of *TPK1* in the respective deletion mutant strain has restored NHEJ efficiency to near wild-type. In contrast, neither *tpk2/3* nor *bcy1* mutants showed comparable defect (Figure 1B). When combined with *tpk2/3*, or *bcy1* mutants, *tpk1* deletion did not display additional reduction in DSB repair when compared with *tpk1* mutant alone. This suggests a role for Tpk1 in NHEJ, irrespective of other PKA catalytic and regulatory subunits.

Since plasmid-based DSB can be functionally distinct from chromosomal DSBs (20), we created *tpk1-3* and *bcy1* mutants in the yeast parental JKM139 strain, which in the presence of galactose expresses the HO-site specific endonuclease under the galactose or *GAL1* promoter, causing *in vivo* chromosomal DSBs and producing 3′ overhangs at *MAT* locus (27). Since JKM139 strain lacks two silent copies of the *MAT* locus (*HML* and *HMR* homologous regions) that would be required to repair HO-induced DSBs by HR, efficient repair of DSBs will thus rely on NHEJ (27). The rate of cell survival was estimated for each mutant by the number of colonies formed on galactose as opposed to colonies grown on glucose-supplemented media. As with the plasmid repair assay (Figure 1B), when DSB was induced with galactose, the *tpk1* mutant significantly reduced (*P* ≤ 0.05) cell survival, and further reductions in the *yku70* NHEJ control was observed as expected, but not for *tpk2*, *tpk3* or *bcy1* mutants (Figure 1C). To examine if the loss of *tpk1* explicitly impacts NHEJ and not HR, we performed an HR plasmid repair assay (28), and found that *tpk1* mutant did not impair HR (Figure 1D), as compared to the HR-defective control *rad54*.

To further examine whether repair patterns are altered in the *tpk1* mutant when compared with wild-type due to intrinsic changes pertaining to *tpk1* deletion, at least 24 independent colonies from the plasmid repair assay were isolated and subjected to sequencing to identify mutagenesis rates at the site of DNA DSB repair. While occasional mutagenesis was observed in wild-type cells (i.e., 90% of the plasmids recovered showed intact cleavage sequence, with 10% showing nucleotide loss of either >4 or <4 bp), 75% of the plasmids recovered from the *tpk1* mutant showed >4 nucleotide loss at the cleavage site, which was comparable to plasmids recovered from the *yku70* mutant (Figure 1E), indicating substantial resection at the breakage site for each deletion mutant, consistent with less efficient NHEJ repair events.

### Tpk1 is functionally related to Nej1 and the Dun1 checkpoint kinase

**Figure 2.**
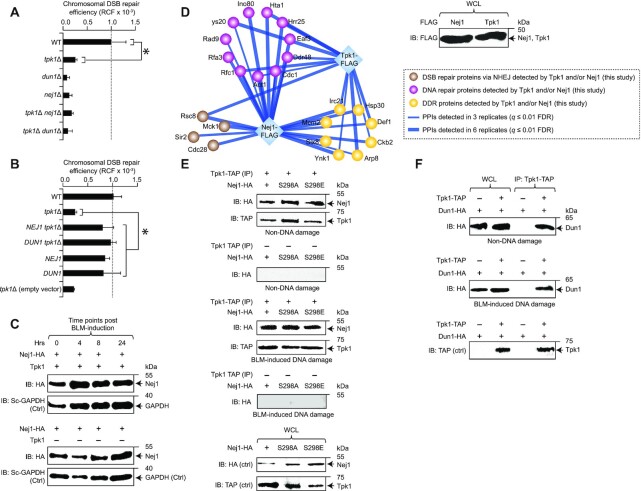
Tpk1 effect on NHEJ is associated with Dun1 and Nej1. (**A, B**) Chromosomal DSB repair efficiency in *tpk1* and *dun1* (or *nej1*) double mutants (A) and their single mutants, as well as in the overexpression (B) of *NEJ1* or *DUN1* in *tpk1* mutants. (**C**) Nej1 protein expression at different time points post BLM-induction in HA-tagged Nej1 in wild-type (WT) and *tpk1* mutants along with yeast (Sc) GAPDH control (Ctrl). (**D**) Immunoblot (IB) showing the native expression of chromosomally FLAG-tagged Tpk1 and/or Nej1 in whole cell lysates (WCL) and their physical association with proteins (filtered at *q* ≤ 0.01 false-discovery rate, FDR) involved in DNA repair-related processes (Supplementary Table S3); PPIs, protein-protein interactions. (**E, F**) TAP-tagged Tpk1 immunoprecipitated (IP) with calmodulin beads from the cell extracts of HA-tagged Nej1 (wild-type, mutagenized; E) or Dun1 (F), treated with or without bleomycin (BLM) in the presence or absence of Tpk1, was IB’ed with anti-TAP or anti-HA antibody. The expression of TAP or HA-tagged proteins in whole cell or IPed lysates were IB’ed with anti-TAP or anti-HA antibody as controls (ctrl). Molecular masses (kDa) of marker proteins are shown in C, E and F. Data in panels A and B represent mean ± SD (*n* = 5 biological replicates; *P ≤ 0.05 by Student's *t* test).

The previous identification of Nej1 as a potential substrate for Tpk1, and Dun1’s established role in phosphorylating Nej1 as part of the NHEJ repair (12,17) suggest that Tpk1’s role in DSB repair by NHEJ may be functionally reliant on Nej1 and Dun1. We thus probed the effect of *tpk1* and *nej1* or *dun1* single and double mutants on DSBs using the HO-induced chromosomal DSB assay, and found that the *tpk1 nej1* (or *tpk1 dun1*) double mutants showed no further reduction in the DSB repair efficiency compared to the loss-of-function of *tpk1 or nej1* (or *dun1*) alleles alone (Figure 2A). This result suggests that Tpk1, Nej1 and Dun1 co-function in the same pathway.

We next determined whether reduced NHEJ repair efficiency in the *tpk1* mutant can be rescued by the overexpression of *NEJ1* or *DUN1*. Strikingly, *NEJ1* or *DUN1* overexpression from *GAL1* promoter in the *tpk1* mutant has restored chromosomal DSB repair efficiency to near wild-type levels. This rescued phenotype of *tpk1* deletion is compensatory only by *NEJ1* or *DUN1* overexpression, as neither the empty vector in *tpk1* mutant nor *NEJ1* or *DUN1* overexpression in wild-type had effect on DNA repair efficiency (Figure 2B). Overexpression of other core NHEJ genes *in trans* also does not rescue DSB repair deficiency in *tpk1* mutant under HO induction of chromosomal break (Supplementary Figure S1A). Further, we examined the possibility of Nej1 protein levels being regulated by *TPK1* expression after DNA damage, which may explain recovery in the *tpk1* mutant by *NEJ1* overexpression. However, comparable protein level of Nej1 at various time points after bleomycin (BLM, a DSB-generating agent)-induction remained consistent in the wild-type or *tpk1* null strain (Figure 2C). Since these results reveal that the *tpk1* mutant phenotype can be rescued by *NEJ1* or *DUN1* overexpression, and that *TPK1* is not a regulator of Nej1 expression, we conclude that Nej1/Dun1 likely function downstream of Tpk1.

To examine potential physical associations between Tpk1, Nej1 and Dun1, we chromosomally tagged Tpk1 and Nej1 with 5× FLAG-epitope, and the respective proteins were affinity purified to near physiological conditions, independently six times, and further subjected to MS analysis. Interacting proteins filtered at 1% (*q* ≤ 0.01) false-discovery rate from the MaxQuant search engine, and those present in at least three of the six replicates in either Tpk1 or Nej1 purifications, but not in the untagged control strain, were considered high-quality interactions (Figure 2D). Our analysis captured known associations from literature (e.g. Tpk1-Tpk2/Bcy1; data not shown), and additionally found Tpk1 and/or Nej1 to engage in previously unreported interactions implicated in DNA-repair related processes (Supplementary Table S3), including DDR (Slx8), DNA repair (Act1) and NHEJ (Rsc8). The MS experiments have reproducibly co-purified Nej1 with Tpk2 and Bcy1, and not with Tpk1 (data not shown), which could be attributed to detergent-mediated disruption of low abundance or weak binding partners. However, when co-immunoprecipitation was carried out with the use of exogenous expression of HA-tagged Nej1 and chromosomal TAP-fusion of Tpk1, as well as the lysis of cell extracts with no detergent in buffer conditions and gentle cell disruption using glass beads, we found Nej1 to co-precipitate with Tpk1 in cell extracts treated with or without BLM (Figure 2E). Since Dun1 regulates DNA repair pathways (29), we performed co-immunoprecipitation to verify whether Tpk1 and Dun1 interact, with or without DNA damage. We found that Tpk1 co-precipitates with Dun1 (Figure 2F), as with Nej1, which strengthens the possibility of Tpk1–Nej1–Dun1 in the assembly of a macromolecular complex, irrespective of DNA damage induction.

### Tpk1-Nej1 function in parallel to Yku80, to promote repair/prevent DNA resection

**Figure 3.**
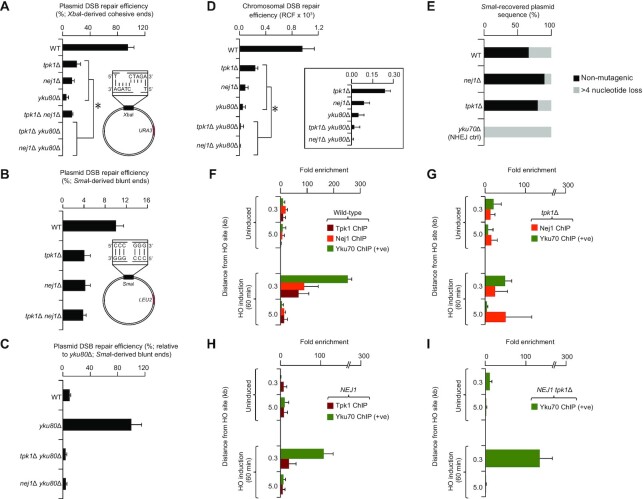
Recruitment of Tpk1 to DNA damage sites. (**A**–**C**) Plasmid repair efficiency of DSBs with cohesive (A) and blunt (B, C) ends, where *tpk1* (or *nej1*) *yku80* double mutants were shown relative to *yku80* (C) single mutant due to its increased repair efficiency of blunt end breaks as reported (32). (**D**) Chromosomal DSB repair efficiency of *tpk1* double and single mutants is shown as a zoom-in. (**E**) *Sma*I- recovered plasmid mutagenesis (*n* ≥ 20 clones) from wild-type (WT) and mutants. (**F–I**) Fold enrichment of Tpk1, Nej1 and/or Yku70 (positive control) in WT (F), Nej1 overexpression (H), or *tpk1* mutant (I) with or without (G) Nej1 overexpression, before and after HO-induced DSB (i.e. from 0.3 and 5.0 kb distance from the damage site, along with control locus). Data are mean ± SD (*n* = 5 biological replicates; *P ≤ 0.05 by Student's *t* test).

The Yku heterodimer (Yku70, 80) is critical for the repair of XbaI-produced cohesive ends through NHEJ, but dispensable for the repair of SmaI-generated blunt (non-cohesive) DNA DSBs due to MMEJ (30). We therefore examined the possible functional crosstalk between Tpk1 and Yku80 in NHEJ and MMEJ. Strikingly, deletion of both *tpk1* and *yku80*, or *nej1* and *yku80* mutants severely impaired the ability of yeast cells to repair XbaI-cut cohesive DNA DSBs, resulting in mere efficiency of 0.03% for the *tpk1 yku80*, as recently reported (18), and 0.17% for the *nej1 yku80* double mutants when compared to single mutants (Figure 3A).

In regard to SmaI blunt-ended DNA DSBs, *tpk1* or *nej1* mutants exhibited reduction in efficient repair (4.0% for *tpk1*, 4.2% for *nej1*, Figure 3B) when compared to an already inefficient wild-type (10%) as reported (31). While no further decrease in DSB repair efficiency with blunt ends was noticed in the *tpk1 nej1* double mutants (Figure 3B), the digenic combination of *yku80* with *tpk1* (or *nej1*) deletions has instead led to an additional reduction in DSB repair efficiency (4.1% for *tpk1 yku80;* 4.4% for *nej1 yku80* double mutants) with respect to *tpk1* or *yku80* single mutants. Notably and in a marked contrast to *tpk1*, the *yku80* single mutant is highly proficient in repairing *Sma*I-cleaved blunt-ended DSBs when compared to wild-type (Figure 3C), consistent with reports (18,32), indicating Ku's role in the suppression of error-prone repair mechanisms. In any case, our results suggest that Tpk1 and Nej1 operate parallel to Yku80 in lesion repair of short cohesive 5′ overhangs, while being significant for the repair of blunt-ended DNA lesions, implying a shared role in NHEJ and MMEJ. Consistent with this, we found that deletion of *tpk1* or *nej1* in the *yku80* mutant led to further reduction in repairing DSBs (Figure 3D).

To further examine the functional role of Tpk1 in relation to Nej1 and Yku80, we characterized plasmid repair events from transformed colonies by recovery of the plasmid and sequencing. Contrary to the repair of cohesive-ended DSBs (Figure 1E), but consistent with previous finding (10), we found that *nej1* deletion led to a decrease in mutagenesis of plasmids recovered from transformed colonies (i.e. 10% of the plasmids show >4 nucleotide loss at the cleavage site), likely due to reduced end processing activities of Rad27 stimulated by Nej1 (10). In the repair of *Sma*I blunt-ended DSBs, the *tpk1* mutant resulted in less mutagenesis (i.e., 20% of plasmids show >4 nucleotide loss) than wild-type, resembling more closely the *nej1* mutant, whereas *yku80* mutant, as expected, resulted in increased mutagenesis (Figure 3E). This implies that Tpk1 function is in alignment with Nej1 than Yku80 in NHEJ-mediated repair.

Next, the recruitment of Tpk1, Nej1 and Yku70 to the DSB site was investigated by performing ChIP, followed by qRT-PCR using primers designed for proximal to and more distal from the expected site of DNA damage, 0.3 and 5.0 kb of the HO cleavage site at the *MAT* locus (Supplementary Figure S1B). Specifically, ChIP was conducted with an HA-specific antibody probing for an HA-fusion tag on the C-terminal end of Nej1, and in parallel utilized a protein-specific antibody to perform Tpk1 or Yku70, in wild-type JKM139 cells, before and after 60 min of HO-induced DSBs. Our data showed significant enrichment of DNA from Tpk1 immunoprecipitates within 0.3 kb from the site of damage within 60 min of HO induction, while no increase was observed at 5.0 kb from the cleavage site when compared to uninduced cells (Figure 3F), suggesting that Tpk1 is recruited to the DNA break site as early as 60 min after HO induction. Similarly, Yku70 and Nej1 also were highly enriched at 0.3 kb from the HO cleavage site compared to uninduced cells, and not at more distal locations or the control locus.

In *tpk1* mutants however, Yku70 immunoprecipitated with DNA to a much lower degree at 0.3 kb from the cleavage site over the uninduced control (Figure 3G), implicating either a reduced recruitment or retention at the break site. Further, enrichment of DNA from Nej1 was diminished at 0.3 or 5.0 kb distant from the DSB (Figure 3G), suggesting a reduction of Nej1 recruitment in the absence of *tpk1* or a delay/disruption in the Nej1 recruitment due to Yku70 loss, and potential DNA-end resection. Since overexpression of *NEJ1* recovers chromosomal DSBs in *tpk1* mutant (Figure 2B), we performed Yku70 or Tpk1 ChIP from cells overexpressing *NEJ1* from the same GAL1 promoter inducing HO cleavage. In contrast to uninduced cells, *NEJ1* overexpression resulted in similar Yku70 and Tpk1 enrichment of DNA at 0.3 kb from the break site as with the wild-type (Figure 3H), consistent with normal repair processes. When *NEJ1* was overexpressed in a *tpk1* mutant, we observed the recovery of DNA, 0.3 kb from breakage of Yku70 (Figure 3I), suggesting that *NEJ1* overexpression contributes to the reduction of large sequence DNA-end resection, and restores Yku70 close to the break site to wild-type levels.

### Phosphoproteomics identify the Nej1 phosphorylation site of Tpk1

Previous large-scale phosphorylation mapping of yeast (17) has putatively suggested Nej1 as potential substrate for protein serine/threonine kinases (Yck1, Atg1), AMP-activated serine/threonine kinase (Snf1), calmodulin-dependent protein kinase (Cmk1), and the cAMP-dependent protein kinase catalytic subunit (Tpk1) under non DNA-damaging condition. Given the native physical association between Nej1 and Tpk1, we investigated whether Nej1 is being targeted by Tpk1 in the presence or absence of HO-induced DNA damage. Endogenously expressed FLAG-tagged Nej1 was purified from wild-type and *tpk1* null strains, independently in triplicate, before and after HO-induced DSBs. Phosphopeptides from protein extracts enriched using titanium dioxide-metal oxide affinity chromatography were analyzed by MS for peptides phosphorylated (*q* ≤ 0.05) in wild-type and *tpk1* mutant (Supplementary Figure S2A). Phosphosites were identified by applying a post-translational modification site-specific probability-based cut-off of ≥25% based on known phosphosites from literature/public databases, and retaining phosphosites detected in 2 of 3 replicates or found in at least ≥ 2 phosphopeptides under any given condition.

In total, 33 phosphorylation sites on Nej1 were identified, of which 25 were Tpk1-independent (i.e. uncovered in *tpk1* mutant and seen or lost in wild-type cells) and 8 Tpk1-dependent (i.e. identified in wild-type cells but lost in *tpk1* mutant) in HO-induced DSBs (Supplementary Figure S2B, Supplementary Table S4). Among the latter, S297/298 were previously reported as Nej1 phosphorylation sites in response to DNA damage, and targeted by Dun1 as part of its NHEJ repair mechanism (12). Also, Nej1 sequence alignment revealed S297/298 and a putative conserved Nej1 phosphorylation motif ‘KPKSRE pS pST’ located at S297/298 with known relatives or in different strains of yeast, suggesting an evolutionary conserved role for these sequences (Supplementary Figure S1C). Two-thirds (21, 64%; Supplementary Figure S2B) of Tpk1-dependent/independent phosphorylations occurred on Nej1 serine residues. Six phosphosites (Y53/131,S58/297/298/323) identified in yeast Nej1 were also conserved in the human XLF (Supplementary Table S4), and nearly half of the yeast Nej1 residues (15–179 position) exhibited a consensus for an XLF domain (Supplementary Figure S2C).

Among the serine phosphosites detected on Nej1, 14 were unique to uninduced or HO-induced DSBs, while 7 were shared across conditions (Supplementary Figure S2B). For instance, S294 phosphorylation on Nej1 was dependent on *tpk1* in both non-DNA damaging and DNA-damaging conditions, whereas *tpk1*-dependent phosphorylation at S301 was observed only in the non-DNA damaging condition. Conversely, the *tpk1*-independent S111 phosphosite of Nej1 under non-DNA damaging condition suggests that phosphorylation is possibly induced by other kinases without DNA damage. Also, *tpk1*-independent S74/76 phosphorylation on Nej1 under HO-induced DSBs, including the previously highlighted (12) DDR kinase phosphorylation sites in Nej1 (S63/68, T60), may represent modifications vital to coordinating cellular responses to DNA damage that are in some way abrogated by Tpk1 to potentially regulate DNA repair.

### Tpk1-mediated Nej1 S298 is vital for nuclear localization and NHEJ repair

Tpk1 preferentially targets R-[K/R]-X-S sequences (17), with two R-X-X-S matching sequences approximating this recognition site within Nej1, including at the phosphorylated S298. This prompted us to consider S298 of Nej1 as potential Tpk1 phosphosite, which we have tested in using non-phosphorylatable (i.e. mutagenizing S298 to alanine (S298A) in the wild-type to mimic dephosphorylation) and phosphomimetic (i.e. modifying S298 to glutamate (S298E) to mirror constitutive phosphorylation) mutants. Tpk1 association with Nej1 was observed in both S298A or S298E mutants under DNA damaging or non-DNA damaging conditions (Figure 2E), suggesting that Tpk1 is physically coupled with Nej1, independent of the phosphorylation state or DNA damage. The kinase activity assay performed using purified Tpk1 and Nej1 led to strong kinase-dependent consumption of ATP (i.e. a phosphorylation effect) when compared to Tpk1 or Nej1 protein alone (Supplementary Figures S2D and E). Notably, the kinase activity of Tpk1 was impacted in Nej1 S298A and S298E variant strains (Supplementary Figure S2E).

**Figure 4.**
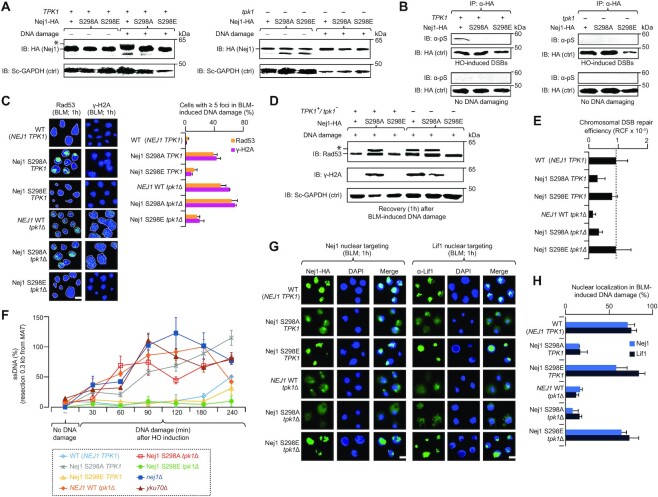
Tpk1 phosphorylates yeast Nej1 at S298. (**A, B**) Extracts from the Nej1-HA wild-type (WT) and mutant strains harbouring with (+) or without (−) Tpk1 in the presence (+) or absence (−) of HO-induced DSBs were immunoblotted (IB’ed) with anti-HA antibody to detect DNA-damage induced mobility shift of phosphorylated Nej1 (*; A) or with anti-phosphoserine (B) antibody to identify phosphorylated serine. Yeast (Sc) anti-GAPDH (A) or anti-HA (B) were used as a control (Ctrl). (**C**) Rad53 and γ-H2A foci (*n* = 200 cells per sample) in BLM-induced WT and *tpk1* mutants, with or without mutagenized Nej1, immunostained with anti-Rad53 or anti-γ-H2A antibody. Nuclei stained with DAPI are indicated with dotted lines. Scale bar, 5 μm. (**D**) Phosphorylated Rad53 (*) and H2A in the indicated WT and *tpk1* mutants, with or without Nej1 variant, after 1 hr of recovery from BLM induction was analyzed by probing with anti-Rad53 and anti- γ-H2A antibody, along with yeast (Sc) anti-GAPDH as a control. (**E**) HO-induced chromosomal (RCF, relative colony formation) DSBs on endogenously expressed Nej1 WT or variants in the presence or absence of *tpk1*. (**F**) Accretion of resected ssDNA in the strains with no DNA damage or recovered after HO induction (i.e. 0.3 kb from break site) at varying times points. (**G, H**) Micrographs (G) and quantification (H; *n* = 100 cells per sample) of Nej1 and Lif1 nuclear targeting in the BLM-induced Nej1 WT or variants, carrying with or without *tpk1*, after probing with anti-HA or anti-Lif1 antibody. DNA stained with DAPI. Scale bar, 5 μm. Data for E and F are mean ± SD (*n* = 3 biological replicates; *P ≤ 0.05 by Student's *t* test). Molecular masses (kDa) of marker proteins are indicated in panels A, B and E.

To validate Nej1 S298 site as the direct target of Tpk1 activity, we examined electrophoretic mobility of Nej1 changes in the absence or presence of DNA damage (i.e. HO-induction) with chromosomally integrated Nej1-HA in wild-type or *tpk1* deleted cells, with or without mutation (S298A, S298E). In contrast to wild-type, DNA-damage induced mobility shift was lost in cells producing Nej1 variants. In *tpk1* deletion, no Nej1 mobility shift changes were detected in wild-type Nej1 or variants (Figure 4A). This was confirmed by immunoprecipitating Nej1 from wild-type and *tpk1* deleted cells, with or without Nej1 variants, in the presence or absence of DNA damage, followed by immunoblotting with phosphoserine antibody. In line with other findings, wild-type Nej1 was phosphorylated in DNA damage induced cells containing Tpk1, but phosphorylation was halted for Nej1 variants from wild-type *TPK1* or *tpk1* deletion (Figure 4B).

Next, the potential relevance of S298 to DNA repair was monitored by comparing nuclear foci formation at DSB sites using surrogate markers (Rad53, γ-H2A) of DNA damage (20) in *NEJ1* wild-type and variants in *tpk1* null and wild-type background strains treated for 1 hr with DSB*-*inducing drug, BLM. Both Rad53 and γ-H2A foci were increased in the Nej1 S298A variant strain in wild-type *TPK1* or *tpk1* deletion after BLM treatment, mimicking the *tpk1* mutant alone. Conversely, the Nej1 S298E variant in wild-type *TPK1* or *tpk1* deletion has emulated wild-type cells (Figure 4C), suggesting that loss of *tpk1* had minor effect on foci formation in phosphomimetic Nej1 at site 298. These findings are in line with the Rad53 and γ-H2A phosphorylation levels detected 1 hr post-BLM-induced DNA damage by immunoblotting in wild-type *TPK1* or *tpk1* deletion mutant producing the Nej1 S298A variant (Figure 4D), while dephosphorylation of Rad53 and γ-H2A levels were noted in the wild type *TPK1* or *tpk1* deletion mutant producing the Nej1 S298E variant. We then examined whether Tpk1 was essential for Nej1 phosphorylation at S298 in NHEJ resolution. Chromosomal NHEJ repair with endogenously produced S298E-mutagenized Nej1 in the *tpk1* mutant showed that Nej1 S298E in the *tpk1* mutant restored NHEJ efficiency comparable to wild-type, while S298A exhibited a profound repair defect (Figure 4E), indicating that S298 of Nej1 contributes to the recovery of NHEJ defect in *tpk1* mutant, consistent with S298 requirement for efficient NHEJ (12).

To examine resection at the DNA repair site in *tpk1* mutant or Nej1 variants, we isolated chromosomal DNA from JKM139 strains under constitutive HO induction of DNA damage at multiple time points over a 4 hr time course and subjected the isolated DNA to *Psi*I restriction digestion, which cuts dsDNA in the region amplified by qRT-PCR between the primer pairs at 0.3 and 5.0 kb distal from HO cleavage. QRT-PCR amplification is thus dependent on a failure of PsiI digestion, indicative of ssDNA due to resection, and a proportion of qRT-PCR amplification versus undigested control generates a percent resection at the corresponding locus. Wild-type cells exhibited minor resection at late time points (4 h) consistent with repair cycles occurring efficiently and minimal resection, however, as with a failure of NHEJ and resection by *yku70* or *nej1* mutants, we found an increase in resection starting at 30 min and continuing at all time points proximal to the break site, with resection at the 5.0 kb locus emerging later (Figure 4F, Supplementary Figure S2F). While *tpk1* mutant did not exhibit the same degree of resection at 30–60 min, by 90 min resection was comparable to *nej1* mutant at 0.3 and 5.0 kb.

Both Nej1 S298A and *tpk1* mutant producing the Nej1 S298A variant exhibited similar behavior, suggesting that ablation of S298 phosphorylation is similar to the *tpk1* mutant, while the phosphomimetic Nej1 S298E and *tpk1* mutant producing Nej1 S298E variant showed reduced resection at all time points (Figure 4F, Supplementary Figure S2F). This suggests that the Nej1 S298E variant can recover NHEJ defect, and the downstream impacts of *tpk1*-deficient mutant failure to repair DSBs results in DNA resection. Tpk1 acts through Nej1, which stimulates resection via Rad27, and prevents more extensive resection (22). Together, we conclude that S298 phosphorylation through Tpk1 activity is needed for efficient NHEJ repair, and the failure of this repair due to the loss of Tpk1 activity or S298 phosphorylation leads to DNA resection, and consequently adopting MMEJ as an alternate repair strategy.

Previous reports show the C-terminus of Nej1 to be critical for Nej1 localization to the nucleus, its interaction with Lif1, and NHEJ repair (33). These observations and Nej1’s function as part of the DNA ligase IV complex (Dnl4-Lif1), led us to determine whether *tpk1* mutant or Nej1 variants impact Nej1 or Lif1 localization to the nucleus. We found that the *tpk1* mutant and Nej1 S298A variant produced in wild-type or *tpk1* mutant resulted in a loss of Nej1 or Lif1 localization in the nucleus, with (Figure 4G and H) or without (Supplementary Figures S3A and S3B) DNA damage, whereas nuclear localization was restored in cells harboring the wild type or *tpk1* mutant producing the Nej1 S298E variant, suggesting that both Tpk1 and Nej1 S298 are important for nuclear targeting, in addition to NHEJ repair (Figure 2A).

### The yeast Tpk1 role in NHEJ break repair is conserved in mammalian cells

**Figure 5.**
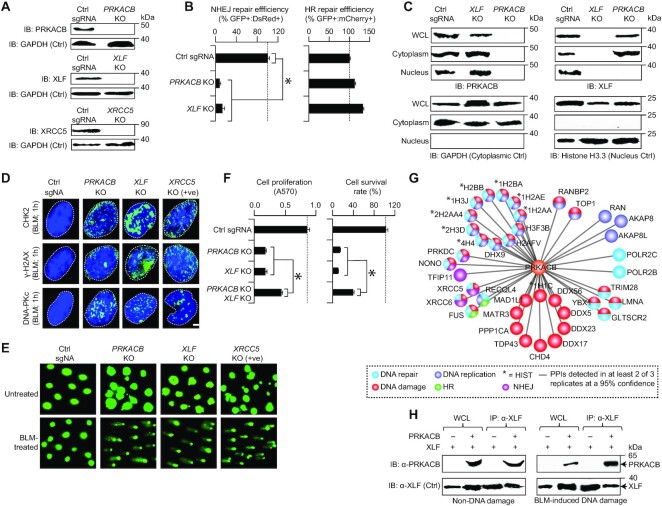
PRKACB, a human homolog of TPK1, is required for NHEJ. (**A**) Immunoblotting (IB) of endogenous PRKACB, XLF, or XRCC5 levels in control sgRNA (Ctrl sgRNA) and knockout (KO) U2OS cells with protein-specific antibody. GAPDH serve as a control (Ctrl). (**B**) NHEJ or HR repair efficiency of *PRKACB* or *XLF* KOs normalized to the control sgRNA was measured based on the ratio of GFP^+ve^ : DsRed^+ve^ to DsRed^+ve^ or GFP^+ve^ : mCherry^+ve^ to mCherry^+ve^ cells. (**C**) PRKACB or XLF localization in the whole cell lysates (WCL), as well as in the cytoplasmic and nuclear extracts of Ctrl sgRNA and *PRKACB* or *XLF* KOs probed with anti-PRKACB or anti-XLF antibody. GAPDH for cytoplasmic and Histone H3.3 for nucleus was used as controls. (**D**) CHK2, γ-H2AX, and DNA-PKc foci (*n* = 200 cells per sample) in control sgRNA and KO U2OS cells in response to BLM were immunostained with anti-CHK2, γ-H2AX, and DNA-PKc antibody. Nuclei stained with DAPI indicated in dotted lines. Scale bar, 20 μm. (**E**) Alkaline comets of Ctrl sgRNA or KO U2OS cells treated with or without BLM. (**F**) Proliferation and survival of control sgRNA and KO U2OS cells. (**G**) PRKACB interacting proteins (filtered at a 95% confidence from MS) in DNA repair-related processes; PPIs, protein-protein interactions. (**H**) XLF immunoprecipitates and input whole cell lysates (WCL) from PRKACB WT and KO U2OS cell extracts treated with or without BLM were IB’ed with PRKACB antibody, along with XLF as a control (Ctrl). Data for B and F are mean ± SD (*n* = 3 biological replicates; *P ≤ 0.05 by Student's *t* test). Molecular masses (kDa) of marker proteins are indicated in panels A, C and H.

Given that the yeast PKA catalytic subunits (Tpk1-3) share 47–49% sequence identity with their human PRKACB counterpart (3e–127 ≤ *E*-value ≤ 7e–121; Supplementary Figure S3C), and the yeast Nej1 human homolog, XLF, facilitates NHEJ by bringing together broken DSB ends (34), we examined whether PRKACB and XLF display similar NHEJ activity in human U2OS osteosarcoma cells (an *in vitro* model cell line for studying DDR genes (20)). To test this possibility, we performed an *in vivo* end-joining pEGFP-Pem1-Ad2 reporter assay (23), whereby I-SceI digestion results in a DSB being created and successful repair by NHEJ essentially restores GFP expression (Supplementary Figure S4A). As with the yeast *tpk1* and *nej1* (Figure 2A), CRISPR-Cas9 mediated *PRKACB* or *XLF* KOs confirmed by immunoblotting in U2OS cells (Figure 5A) significantly (*P*-value ≤ 3.1 × 10^–3^) reduced GFP and DsRed-positive cells to 8–13%, as opposed to the non-targeting control (Figure 5B). Reduced NHEJ-mediated DSB repair by PRKACB is specific, since *PRKACB* KO in U2OS cells did not exhibit reduction in HR activity as in *XLF* KOs (Figure 5B, Supplementary Figure S4B) with pDRGFP reporter (35), suggesting a conserved role for Tpk1 or Nej1 human homologs in NHEJ repair. Immunoblotting with cytosolic and nuclear extracts of *PRKACB* or *XLF* KOs in U2OS cells using protein specific antibodies further indicated that PRKACB is required for XLF localization to the nucleus (Figure 5C), similar to that of the yeast Tpk1, in both nuclear targeting and NHEJ repair.

To further examine the impact of *PRKACB* deletion on DNA repair, we tested whether mammalian cells can accumulate nuclear CHK2, γ-H2AX and DNA-PKc foci when the depletion of end-joining repair proteins leads to increased DSBs (36–38). As in *XLF* and in the positive control *XRCC5* (homolog of yeast Yku80) KOs, loss of *PRKACB* in BLM-induced DNA damage displayed significant (0 < *P* < 5.0 × 10^−2^) increase in the foci formation (Figure 5D, Supplementary Figures S4C–E), congruent with unrepaired DNA breaks, indicating a reduced capacity for NHEJ repair. This upsurge in foci formation led us to assess whether loss of *PRKACB* in U2OS cells treated with BLM affects DSB repair, using a neutral comet assay. We found that in comparison with BLM-treated control cells, *PRKACB* KO, similar to *XLF* and *XRCC5* KOs, displayed significantly (5.8 × 10^−13^ < *P* < 2.4 × 10^−5^) longer comet tail moment and length, with substantial (2.2 × 10^−3^ < *P* < 3.8 × 10^−2^) increase in comet tail DNA content after BLM treatment (Figure 5E, Supplementary Figure S4F). In agreement with these findings, *PRKACB* or *XLF* KOs significantly (9.5 × 10^−9^ < *P* < 1.2 × 10^−7^) reduced cell proliferation and survival, but was not exacerbated when both *PRKACB* and *XLF* were deleted (Figure 5F). The effect of *PRKACB XLF* double KOs on the survival of U2OS cells to BLM further indicates that the reduction in cell survival was nearly the same as in single KOs (Supplementary Figure S4G), suggesting that PRKACB is likely involved in the same DNA repair pathway as XLF.

Given that genetic interaction pairs are enriched for proteins known to physically interact (26), we immunoprecipitated native PRKACB from U2OS cells and subjected the lysates to MS. After filtering for interacting proteins not present in the control (i.e. cells with no PRKACB antibody), and from two or more replicates at a 95% confidence threshold (*P* ≤ 0.05) from MS, we found 39 high-confidence PRKACB associations (Supplementary Table S5) involving proteins required for NHEJ (e.g. PRKDC, NONO, XRCC5 and XRCC6, a homolog of yeast Ku70) as well as DNA damage (e.g. DDX5/17/23/56 DEAD box RNA helicases) and repair-related functions (Figure 5G). While, as in yeast (Figure 2D), we failed to capture the heterodimeric interaction between XLF and PRKACB by MS, this association was confirmed by co-immunoprecipitation in the cellular extracts of U2OS cells treated with or without BLM (Figure 5H). Together, our data suggest that both, PRKACB and XLF participate jointly in the overlapping NHEJ pathway, and required for efficient repair of NHEJ breaks in U2OS cells.

### Human PRKACB phosphorylation of XLF S263 is vital for NHEJ repair activity

To gain further insights into whether human PRKACB phosphorylates XLF at the same conserved site as in the case of the yeast Nej1 (i.e. yeast Nej1 S298 is equivalent to human XLF S263, Supplementary Figure S5A), we carried out phosphoproteomics by exogenously expressing the FLAG-tagged XLF protein from wild-type and *PRKACB* KO cells in the presence or absence of BLM-induced DSBs. Phosphopeptides enriched with titanium dioxide-metal oxide affinity chromatography were ran in triplicates and analyzed by MS for phosphorylated peptides (*q* ≤ 0.05). After withholding phosphosites detected in 2 of the 3 replicates or those found in at least ≥2 phosphopeptides under any given condition or previously reported in PhosphoSite Plus database, we identified 21 XLF phosphorylated residues in the presence and/or absence of DNA-damaging condition (Supplementary Table S4). Among those are six PRKACB-dependent and eight independent phosphosites in BLM-induced DSBs (Supplementary Figure S5B).

Notably, as with yeast Nej1 S297/298 phosphorylation by Dun1 (12) and Tpk1 (Figure 4A and B), DNA-damage induced PRKACB-dependent phosphosites (T181, 223) that we identified (Supplementary Figure S5C) were previously reported (39,40) as phosphorylated in XLF by a key NHEJ player, DNA-dependent protein kinase (DNA-PKc) and protein kinase B (or AKT), which interact and regulate DNA-PKc to enable the recruitment of repair factors to DSB sites (41). This result suggests that as in yeast, different human kinases cross-talk by targeting the same downstream XLF substrate at the same site for phosphorylation. Sequence alignment of the yeast Nej1 and the human XLF unveiled 10 conserved residues, of which we found only a phosphorylated residue of XLF at S287 and Nej1 at S323 (Supplementary Figure S5A), and not XLF S263, corresponding to the Nej1 S298 phosphosite.

As with yeast, more than half (11, 52%) of the PRKACB-dependent or independent phosphosites are primarily serines, while less than the other half are threonines (9, 43%; Supplementary Figure S5D, Supplementary Table S4). Similar to ATM, ATR and DNA-PKcs (39), yeast Tpk1 or human PRKACB are expected to phosphorylate their substrates on serine or threonine, followed by either glutamine (i.e. SQ/TQ sites) (39,42) or hydrophobic amino acids (43,44) such as leucine (L) or alanine (A). Consistent with this, 13 Nej1 and 7 XLF phosphosites conform to the S/T-hydrophobic consensus (Supplementary Figure S5E, Supplementary Table S4), implying that yeast Nej1 or human XLF are likely substrates for Tpk1 or PRKACB, respectively.

**Figure 6.**
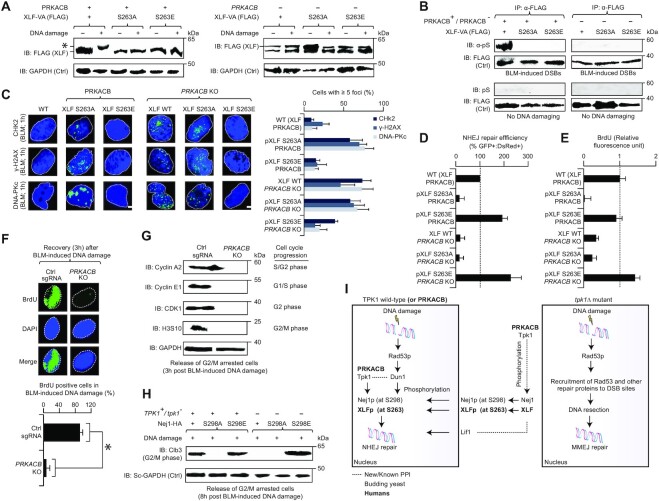
PRKACB phosphorylation of human XLF at S263. (**A, B**) Immunoblotting (IB) of extracts from XLF-VA (containing the FLAG-tag epitope) wild-type (WT) and mutants with (+) or without (−) PRKACB in the presence (+) or absence (−) of BLM were probed with anti-FLAG (A) antibody to detect BLM-induced migration shift of phosphorylated XLF (*) or with anti-phosphoserine (B) antibody to unveil phosphorylated serine. Anti-GAPDH (A) or anti-FLAG (B) were used as a control (Ctrl). (**C**) CHK2, γ-H2AX and DNA-PKc foci (*n* = 200 cells per sample) in BLM-induced WT and *PRKACB* KO, with or without mutagenized XLF, immunostained with anti-CHK2, anti-γ-H2AX, or anti-DNA-PKc antibody. DAPI stained nuclei are indicated with dotted lines. Scale bar, 10 μm. (**D**) NHEJ repair efficiency of XLF WT or variants in U2OS cells carrying PRKACB WT and KO was measured based on the ratio of GFP^+ve^ : DsRed^+ve^ to DsRed^+ve^. (**E**) Immunofluorescence measurement of BrdU incorporation in the strains arrested with double thymidine block and nocodazole, released synchronously into the medium containing BLM, and recovered after 3 hrs from BLM induction. (**F**) BrdU positive cells (*n* = 50 cells per sample) from the immunostaining of strains, after 3 h of recovery from BLM induction with anti-BrdU antibody. DNA stained with DAPI are shown in dotted lines. Scale bar, 10 μm. (**G, H**) Expression of cell cycle regulators in the human (G) and yeast (H) strains after 3 h (G) and 8 h (H) of recovery from BLM induction immunoblotted (IB) with protein-specific antibodies. U2OS cells containing the human strains were synchronized with a double thymidine block, followed by G2/M cell cycle arrest for 11 h by nocodazole, and then released into the medium with BLM. In yeast JKM139-strain cultures, G2/M phase was arrested for 2 h by nocodazole. (**I**) Cellular model showing the yeast Tpk1 role with Nej1 and PRKACB with XLF on NHEJ (left), as well as the loss of *tpk1* in DNA resection and MMEJ (right). See main text for details. Data for D and E are mean ± SD (*n* = 3 biological replicates; *P ≤ 0.05 by Student's *t* test). Molecular masses (kDa) of marker proteins are indicated in panels A, B, G and H.

Since failure to observe the serine phosphorylated residue of XLF at 263 by MS is not indicative of the site not being phosphorylated in the cell (see Supplementary Methods), we reconfirmed the phosphorylation of this residue with a XLF mobility shift, following the treatment of U2OS cells with BLM. To test this, stable U2OS cells were transfected with XLF-FLAG containing serine to alanine (A) and serine to glutamate (E) substitutions of S263 in wild-type or *PRKACB* KO. Notably, as with yeast, changes in XLF electrophoretic mobility in response to DNA damage were observed only in the presence of PRKACB, and not in *PRKACB* KO cells. In S263A or S263E mutants, DNA-damage induced mobility shift of XLF was completely abrogated in wild-type and *PRKACB* KO (Figure 6A). This finding was further confirmed using phosphoserine antibody, where XLF serine phosphorylation was detected only in the DNA damage-treated wild-type cells carrying PRKACB (Figure 6B), but not in XLF S263 mutant transfected with or without PRKACB, prior to or after BLM treatment. We therefore conclude a conserved mechanism whereby DNA-damage induced human XLF S263 phosphorylation is dependent on PRKACB, as with the yeast Nej1 S298 phosphorylation being reliant on Tpk1.

To further determine whether PRKACB-mediated XLF phosphorylation is required for DSB repair *in vivo*, as with yeast (Figure 4C), we examined the formation of nuclear foci at DSB sites by measuring the number of CHK2, γ-H2AX and DNA-PKcs foci in wild-type or *PRKACB* KO stable U2OS cells transfected with or without XLF S263 mutants after the induction of damage by BLM. All three DSB markers showed similar increase in the accumulation of BLM-induced foci by *XLF* S263A mutant in wild-type or *PRKACB* KO cells, same as *PRKACB* KO alone. In contrast, *XLF* S263E in either the wild-type or *PRKACB KO* led to decrease in the number of foci, similar to the wild-type (Figure 6C, Supplementary Figure S5F), indicating that XLF S263 phosphorylation by PRKACB is vital for recruitment to DSBs.

Another test to examine whether PRKACB-dependent S263 phosphorylation of XLF in response to DNA damage regulates NHEJ, we measured NHEJ efficiency using the GFP reporter assay (Supplementary Figure S4A) in wild-type or *PRKACB* KO U2OS cells transfected with or without *XLF* S263 mutants. As with the yeast Nej1 S298 phosphorylation through Tpk1 dependent manner (Figure 4E), we found in human that the *XLF* S263A led to loss of NHEJ efficiency, comparable to *PRKACB* KO, whereas *XLF* S263E did not reduce NHEJ efficiency (Figure 6D). Notably, in a *PRKACB* KO, *XLF* S263A had no additional reduction in NHEJ repair efficiency, but *XLF* S263E recovered the *PRKACB* KO NHEJ defect (Figure 6D). This result further supports that the human XLF S263 and yeast Nej1 S298 are both controlled by the human PRKACB and yeast Tpk1, respectively, to regulate NHEJ activity upon DNA damage.

PKA function in the cell cycle for factors unrelated to NHEJ are established (45,46), and here we examined Tpk1 functionality to NHEJ. Our model implicates the effect of Tpk1 on Nej1 phosphorylation and localization, whereas Nej1 has no known effect on cell cycle progression, mediated by the DDR. We reasoned that Tpk1 could conceivably have other cell cycle regulatory or checkpoint effects independent of the NHEJ regulatory effect, potentially by phosphorylating additional substrates. We thus determined the effect of yeast *tpk1* mutant or Nej1 variants, and the human *PRKACB* KO or XLF variants on the expression of cell cycle regulators in response to DNA damage by immunoblotting, and cell cycle progression using a BrdU incorporation assay.

In human U2OS cells, when both *PRKACB* KO and *XLF* S263A non-phosphorylatable mutants were synchronized at G1/S phase by double thymidine block, and G2/M phase by nocodazole, followed by BLM treatment, we found reduced BrdU incorporation via immunofluorescence detection method (Figure 6E), indicating a delayed progression through S phase. The *XLF* S263E phosphomimetic mutants did not block BrdU incorporation, and therefore resulted in the restoration of wild-type cell cycle recovery in *PRKACB* KO (Figure 6E), suggesting that no additional cell cycle effects beyond delayed DNA repair were seen. This finding is consistent with delayed cell cycle progression and recovery, as *PRKACB* KO U2OS cells immunostained against BrdU antibody, as expected, reduced BrdU incorporation to 7% relative to 92% in the control cells (Figure 6F, Supplementary Figure S6A), and selectively decreased the expression levels of classical cell cycle regulators (Figure 6G, Supplementary Figure S6B). Similarly, when yeast wild type and *tpk1* mutant or Nej1 variants were arrested with nocodazole, followed by treatment with BLM, the G2/M phase mitotic cyclin Clb3 expression showed a loss of cell cycle progression recovery in *tpk1* deletion that was dependent on Nej1 non-phosphorylation, while the phosphomimetic mutation recovered wild-type cell cycle progression patterns (Figure 6H, Supplementary Figure S6C). If Tpk1 or PRKACB exhibited additional roles in the DNA damage cell cycle checkpoint, we would expect deletion of *tpk1* or *PRKACB* to show cell cycle effects regardless of the phosphomimetic mutations. Since that was not the case, cell cycle impairment is likely due to the DNA damage checkpoint, caused by unresolved DSBs because of failed NHEJ rather than any additional Tpk1 or PRKACB effect on cell cycle arrest.

## DISCUSSION

Kinases participate in the regulation of many cellular processes, including DNA damage repair. In yeast, DDR checkpoint kinases such as Dun1, Rad53, Tel1 (ATM homolog) and Mec1 (ATR homolog) are key regulators of DNA repair pathways that play crucial roles in DNA-damage signaling and in directing the cell to a specific repair pathway and recruitment of repair proteins (47). In addition, a previous study in yeast has implicated a role for the cAMP-dependent protein kinase catalytic subunit, Tpk1, in DNA damage checkpoint, and suggests it is a component of the Rad53 response (13), while another suggested a role in the regulation of NHEJ efficiency (18,19). Yet, the mechanism by which Tpk1 operates and its regulatory function on DNA repair activities have not been established. In this study, we report a new mechanistic role for the yeast Tpk1, in NHEJ, by phosphorylation of its binding partner Nej1 in a manner similar to Dun1, thereby facilitating efficient NHEJ break repair and resolution by Nej1 recruitment into the nucleus. The failure of *tpk1* deletion to repair DNA efficiently, resulting in unresolved DNA damage foci where Rad53 and γ-H2A remain bound to the damaged site implicates alternative end-joining repair mechanisms may be involved in lesion repair via end-processing at the DSB site. This is in agreement with our findings of increased DNA resection activity, leading to increased mutagenesis at the break site, and shifts recruitment for NHEJ factors, such as Yku70–Yku80 away from the initial damage until end-joining can resolve elsewhere.

A previous study found that an Nej1 S298A (a Dun1 phosphosite) non-phosphorylatable mutant decreases NHEJ efficiency in a plasmid-based DSB repair assay, with the phosphomimetic (Nej1 S298E) substitution recovering impaired NHEJ activity in *dun1* deletion background (12). Using the same phosphomimetic switch approach, we were able to recover end-joining repair defect in the *tpk1* mutant as well. This is consistent with the S298 residue being directly targeted by both Dun1 and Tpk1, or alternatively that Dun1 phosphorylation at S298 is functioning downstream of Tpk1 on Nej1. Overexpression of Dun1 or Nej1 to recover *tpk1* deletion is similarly consistent with either a model in which Dun1 and Tpk1 share a function, or one in which Dun1 functions downstream of Tpk1. However, our results from the *in vitro* kinase activity with direct Tpk1 effect on Nej1, and the loss of phosphorylation-induced DNA damage-dependent mobility shift of Nej1 S298A mutant strongly indicates that Tpk1 and Dun1 share a kinase activity at S298. Physical recruitment of Tpk1 to Nej1, regardless of phosphomimetic mutation or the presence of DNA damage, indicates that Tpk1 behavior is not dependent on Dun1’s effect on Nej1, and that Tpk1 recruitment to the breakage site denotes this physical interaction as relevant in the context of NHEJ, which is known to recruit the Dnl4–Lif1–Nej1 complex in the final stages of damage repair. It is also evident from our data that the Nej1 S298A mutant in *tpk1* deletion interacts with damaged DNA and gets recruited to repair foci with Rad53 and γ-H2A proteins, implicating a role for S298 of Nej1 in the assembly or disassembly of repair foci.

Notably, our data suggest that Tpk1-dependent phosphorylation of Nej1 S298 in response to DNA damage might be regulating the recruitment of enzymes involved in DSB processing, and interference with such process can reduce NHEJ activity (12). Given that Nej1 prevents excess resection and promotes NHEJ in a manner that is lost in the Nej1 variant V338A at the C-terminus (22), it supports the idea that the C-terminal end of the protein is key to preventing sequence deletion at DSB breakage sites and efficient non-mutagenic repair. Our results thus fit this conception where we show that Nej1 phosphosite, S298, which is localized to the C-terminal domain, exhibits NHEJ effect in chromosomal DSB repair assay in a non-phosphorylatable condition. Because nuclear localization of Nej1 is affected by the C-terminal domain (33), and Nej1 phosphorylation occur in this domain, our findings that Nej1 localization is affected by S298 variant and *tpk1* mutant is reasonable. In this context, Nej1 overexpression can lead to non-specific localization to the nucleus, even in the absence of Nej1 phosphorylation at C-terminal domain, which could explain why Nej1 overexpression rescues NHEJ activity in *tpk1* mutant.

Our MS data suggest a broader set of phosphorylation events taking place in the cell, including several sites known to be phosphorylated based on previous work (Supplementary Table S4). Thus, other phosphosites identified in Nej1 might be targets of phosphorylation by Tpk1 or other checkpoint kinases, a possibility that was reported previously for Dun1 (12). Nevertheless, we chose to focus our attention on S298 as a known contributor to NHEJ. We cannot preclude the possibility that Dun1 and Tpk1 have a more complex and interconnected kinase network than suggested here, in which S298 may be a master regulator and additional phosphorylation effects occur. Alternately, Dun1 and Tpk1 may respond to different stimuli to activate Nej1 via S298 phosphorylation in different contexts. For instance, Dun1 is directly activated by the DDR or Rad53 pathway, whereas Tpk1 responds to cAMP and cell cycle/carbon source, suggesting similar biological functions for both proteins under different cellular stimuli. Additionally, our observation of the S298 phosphorylation or phosphomimetic mutants to be vital for Nej1 and Lif1 nuclear localization implicates that the effect could be either direct or mediated through other phosphorylation sites that S298 may control in indirect fashion, which warrants further investigation. In any case, Tpk1 phosphorylation of Nej1 on S298 in response to DNA damage suggests that this phosphorylation activity contributes to NHEJ repair.

Based on our results, we propose a model where Tpk1 has a specific role in activating Nej1 via phosphorylation at the same S298 site as Dun1. Tpk1, which is exported from the nucleus to the cytoplasm upon PKA activation, induces phosphorylation of Nej1 at S298, irrespective of DNA damage signals, and thereby localizes Nej1 and Lif1 to the nucleus. DNA damage triggers the activation of Rad53 phosphorylation and DDR, which in turn activates Dun1, which is required for Nej1 phosphorylation in the process of NHEJ, suggesting that phosphorylation may be unstable or lost and S298 must be re-phosphorylated by nuclear Dun1 for efficient NHEJ activation. Based on our Tpk1 ChIP experiments, in conditions where Tpk1 is also nuclear-localized, Tpk1 and Dun1 may be more directly redundant (Figure 6I). In the absence of Tpk1 or the S298 phosphorylation site, Nej1 and Lif1 remain in the cytoplasm, and NHEJ is unable to proceed, ultimately leading to DNA resection and MMEJ (Figure 6I). This is consistent with the previous observation (48) that serine or threonine site substitutions are more frequently phosphorylated by different kinases compared to tyrosine. It is well-established that Dun1 is phosphorylated by Rad53 in response to DNA damage (49), and activation of Tpk1 via DDR has been suggested previously (13) to form the Rad53 foci we see in *tpk1* mutants. Thus, DDR activation of Tpk1 would imply phosphorylation sites on Tpk1, which may correspond to known recognition sites for DDR cascade proteins such as Mec1/Tel1, that target [S/T]*Q sequences (50), or Rad53, which targets a more complex site of [L/M/V/I]-x-[S/T/Y]-x-[L/M/V/I], with the +1 site excluding proline (51). However, Rad53 and Tpk1 are synergistic to the DNA-damaging agent, methyl methane-sulfonate (26), suggesting a separate Tpk1 pathway, distinct from Rad53 activation of Dun1. While many likely recognition sites were found in the primary sequence of Tpk1 (Supplementary Figure S6D), whether Tpk1’s activity in Nej1 phosphorylation is dependent on another component of the DDR, or whether this is regulated by a broad cAMP activation as part of a general stress response, remains to be established.

As in yeast, we have provided evidence that the Tpk1 human homolog, PRKACB, is required for NHEJ repair in U2OS cells (Figure 6I), and deletion of *PRKACB* results in the retention of repair proteins (CHK2, γ-H2AX, DNA-PKc) at unresolved DSB sites at later time points. Further, its interaction with Nej1’s human homolog, XLF, facilitates NHEJ by aligning damaged DSB ends (34,52), with no additional reduction in the survival of BLM-treated U2OS cells harboring *PRKACB XLF* double KOs, when compared to either single KO. This suggests their conserved role in NHEJ, and potential function in a similar DDR pathway. Consistent with this, we have found PRKACB to be associated with the phosphorylation of XLF S263 (a homologous site to yeast S298, Figure 6I), suggesting a conserved DNA repair phosphorylation event between yeast and humans. Also, as with NHEJ function of Nej1 S298 phosphorylation by Tpk1 in yeast, XLF S263 phosphorylation by PRKACB is required for NHEJ activity in mammalian cells. Besides the Nej1 S298 or XLF S263, our phosphoproteomics analyses in yeast and human U2OS cells have identified other Tpk1-dependent Nej1 (S294, T318) or PRKACB-dependent XLF (S22, T66/181/223/275, Y218) phosphosites in response to DNA damage. However, more detailed studies will be required to clarify whether these Nej1 or XLF modifications are targets of Tpk1 or PRKACB in regulating NHEJ and DSB repair events.

## DATA AVAILABILITY

Data pertaining to some of the figures presented in the main text are provided in Supplementary Data file. Proteomics data are deposited to the ProteomeXchange Consortium via PRIDE partner repository with the dataset identifier PXD024695 and PXD024768.

## Supplementary Material

gkab585_Supplemental_Files
