# Supplementary material for: The conserved Tpk1 regulates non-homologous end joining double-strand break repair by phosphorylation of Nej1, a homolog of the human XLF
Source: Nucleic Acids Res. 2021 Jul 9;49(14):8145–60. doi: 10.1093/nar/gkab585 (PMC8373142; doi:10.1093/nar/gkab585)
Supplement: gkab585_Supplemental_Files [file gkab585_supplemental_files.zip › Supplementary information-one single file.pdf]

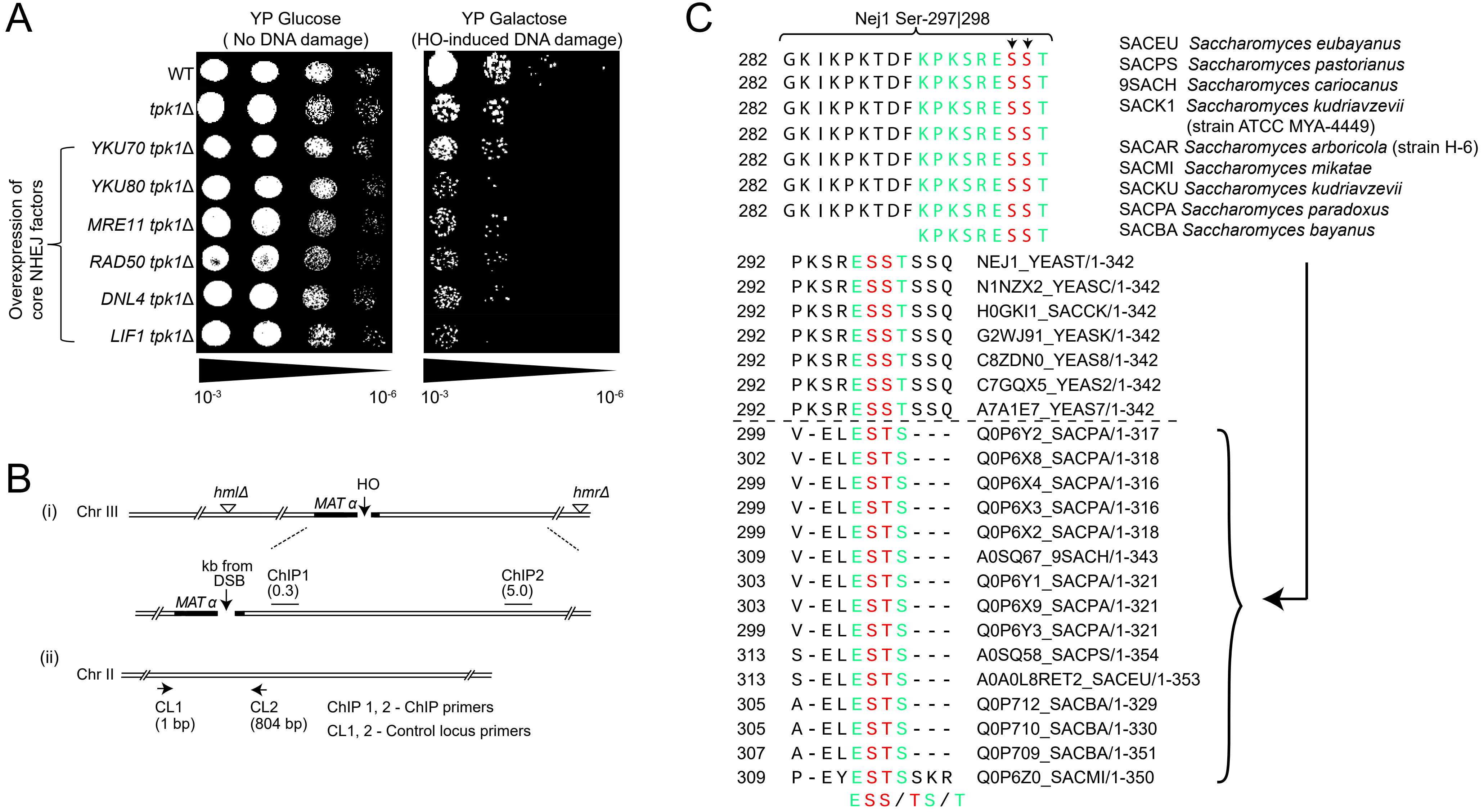

Supplementary Figure S1. Phenotypic rescue of *tpk1* mutant, and evolutionarily conserved Nej1 297/298 residue across different yeast strains. (**A**) Over-expression of core NHEJ genes in *tpk1* deletion mutant in the presence or absence of DNA damage condition. (**B**) Schematic of JKM139 yeast strain on chromosome (Chr) III containing HO endonuclease recognition site, with primer location indicated from the HO cleavage site used for ChIP (i), or at a control (Ctrl) locus (ii) on chromosome II. (**C**) Conserved Nej1 297/298 residue (arrow heads), and a putative Nej1 phosphorylation motif "KPKSRE pS pST" located at Ser-297/298 is shown for closest known relative or different strains of yeast. Abbreviation: 9SACH, *Saccharomyces cariocanus*; YEAS7, *S. cerevisiae* strain YJM789; YEAS2, *S. cerevisiae* strain JAY291; YEAS8, *S. cerevisiae* strain Lalvin EC1118/Prise de mousse;YEASK, *S. cerevisiae* strain Kyokai no.7/NBRC 101557; SACCK, *S. cerevisiae* x *S. kudriavzevii* strain VIN7; and YEASC, *S. cerevisiae* strain CEN.PK113-7D.

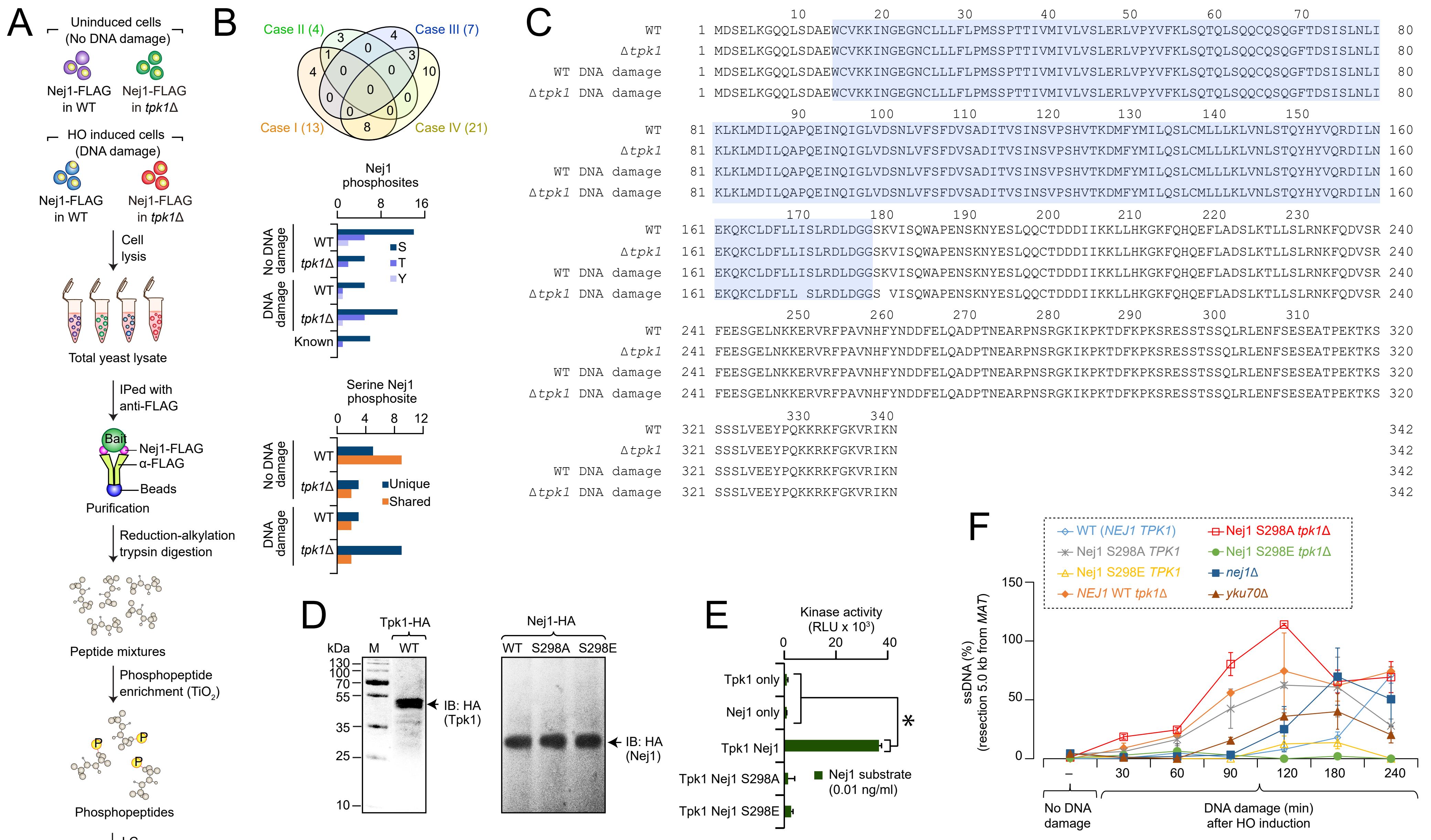

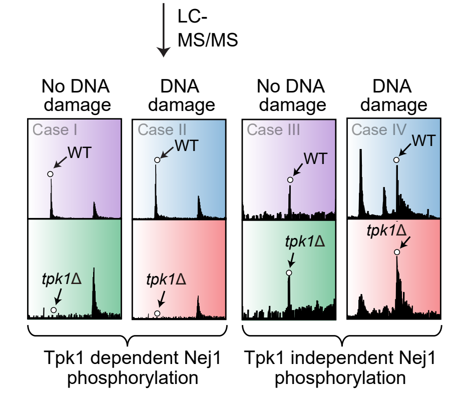

Supplementary Figure S2. Identification of Nej1 phosphorylation site by Tpk1 via phosphoproteomics. (**A**) Phosphoproteomics on chromosomally FLAG-tagged Nej1 in wild-type (WT) and *tpk1* mutant JKM139 cells that are uninduced and induced with HO-endonuclease to create DSBs were immunoprecipitated (IPed) with anti-FLAG, followed by TiO2 phosphopeptide enrichment of the tryptic digested peptides. Mass spectrometry (MS) analysis representing Nej1 phosphosites that are dependent or independent of Tpk1 in non-DNA damage or DNA damage condition. (**B**) Venn diagram (top panel) showing Nej1 phosphosite overlap in each of the Tpk1 dependent or independent cases (see Supplementary Table S5 for details) shown in panel A. Number of Nej1 phosphorylation (STY) sites, including those identified previously in the literature (referred as ‘known’; middle panel), and the most prevalent serine site that are unique or shared (bottom panel) among the indicated strains in DNA damage and non-DNA damaging conditions. (**C**) Yeast Nej1 residue consensus for XLF domain. Half (48%, 164 of 342) of the yeast Nej1 residues (15-179) showing a consensus for XLF domain (XLF-Cernunnos, XRcc4-like factor; blue shade). (**D**) Immunoblotting of HA-tagged Tpk1 or Nej1 (WT or mutagenized) recombinant proteins probed with anti-HA antibody. (**E**) In vitro kinase activity of the indicated recombinant proteins measured based on ATP consumption (RLU, relative luminescence units) at 0.01 ng of Nej1 substrate. (**F**) Accumulation of resected ssDNA in the indicated strains with no DNA damage or recovered after HO induction (i.e., 5.0 kb from the break site) at varying times points. Data for E-F are mean ± SD (n = 3 biological replicates; *$p \leq 0.05$ by Student's *t* test). Molecular masses (kDa) of marker proteins are indicated in panel D.

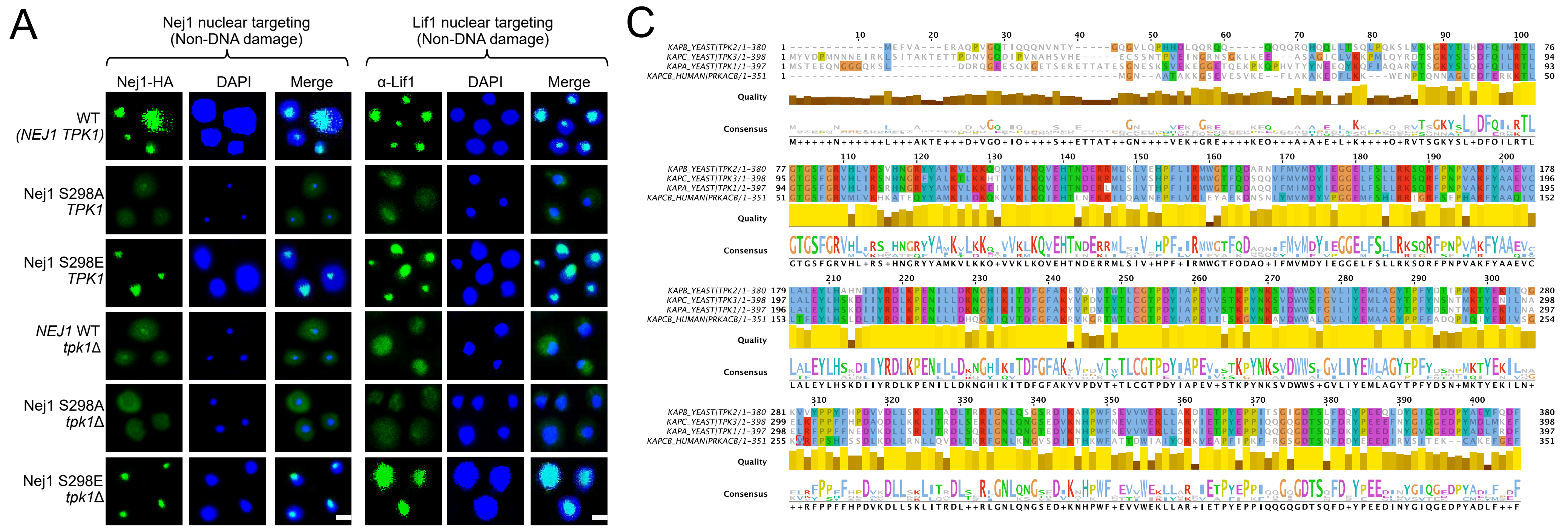

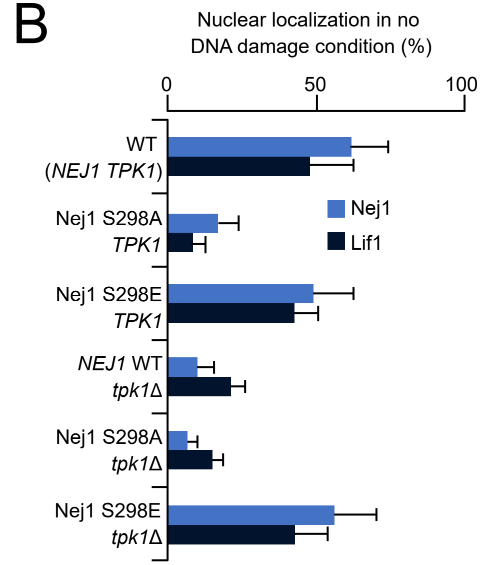

Supplementary Figure S3. Nej1 and Lif1 nuclear targeting, as well as sequence similarity search betwen yeast PKA catalytic subunits and human PRKACB counterpart. (**A, B**) Immunofluorescence micrographs (A) and quantification (B; n = 100 cells per sample) of Nej1 and Lif1 nuclear targeting in the indicated Nej1 wild-type (WT) or variants, carrying with or without *tpk1*, in the absence of DNA damage, after probing with anti-HA or anti-Lif1 antibody. DNA was stained with DAPI. Scale bar, 5 µm. Data for E and F are mean ± SD (n = 3 biological replicates; **p* ≤ 0.05 by Student's *t* test). (**C**) Yeast PKA catalytic subunits (Tpk1-3) sharing sequence identity with human PRKACB.

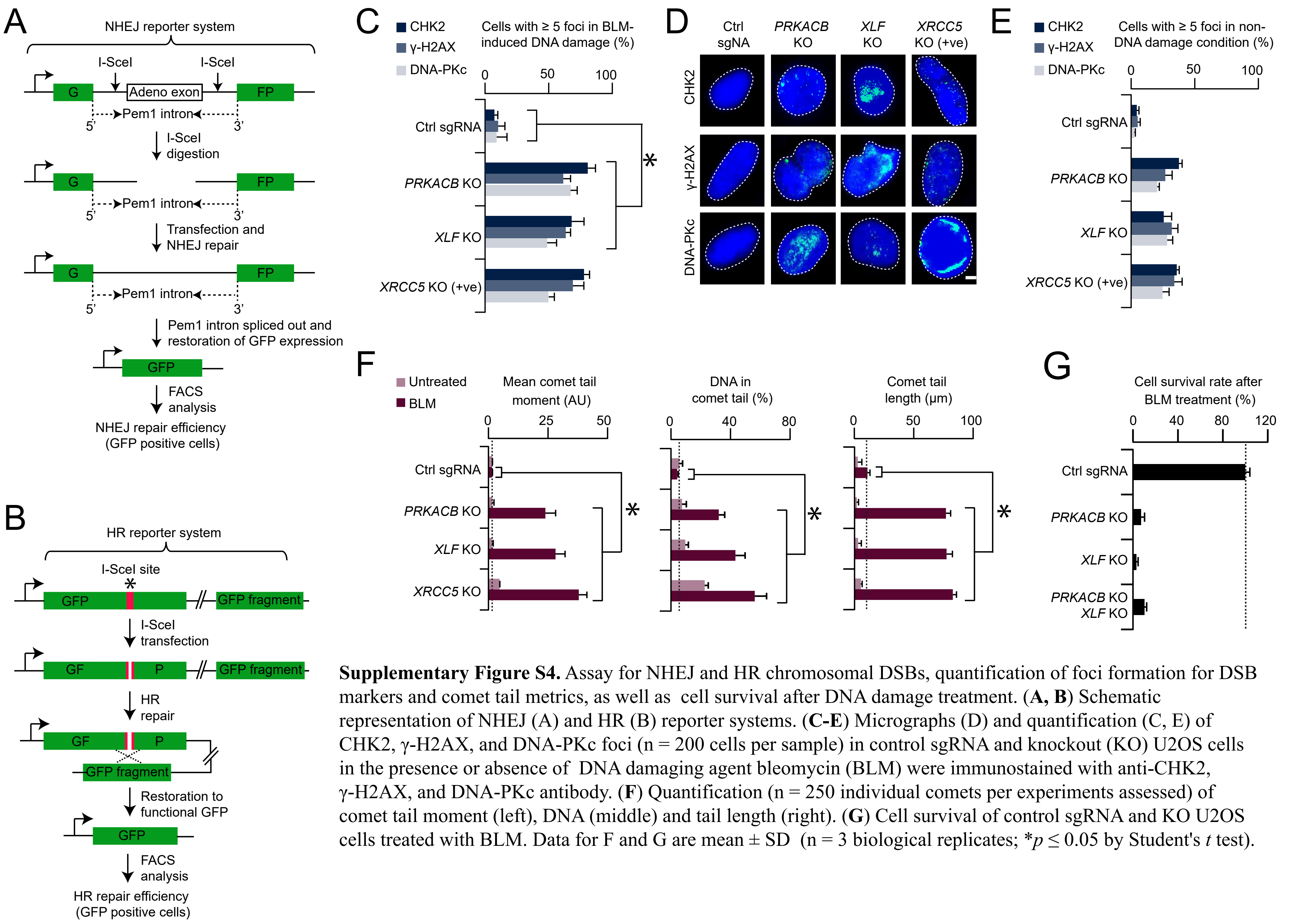

Supplementary Figure S4. Assay for NHEJ and HR chromosomal DSBs, quantification of foci formation for DSB markers and comet tail metrics, as well as cell survival after DNA damage treatment. (**A, B**) Schematic representation of NHEJ (A) and HR (B) reporter systems. (**C-E**) Micrographs (D) and quantification (C, E) of CHK2, γ-H2AX, and DNA-PKc foci (n = 200 cells per sample) in control sgRNA and knockout (KO) U2OS cells in the presence or absence of DNA damaging agent bleomycin (BLM) were immunostained with anti-CHK2, γ-H2AX, and DNA-PKc antibody. (**F**) Quantification (n = 250 individual comets per experiments assessed) of comet tail moment (left), DNA (middle) and tail length (right). (**G**) Cell survival of control sgRNA and KO U2OS cells treated with BLM. Data for F and G are mean ± SD (n = 3 biological replicates; *$p \leq 0.05$ by Student's *t* test).

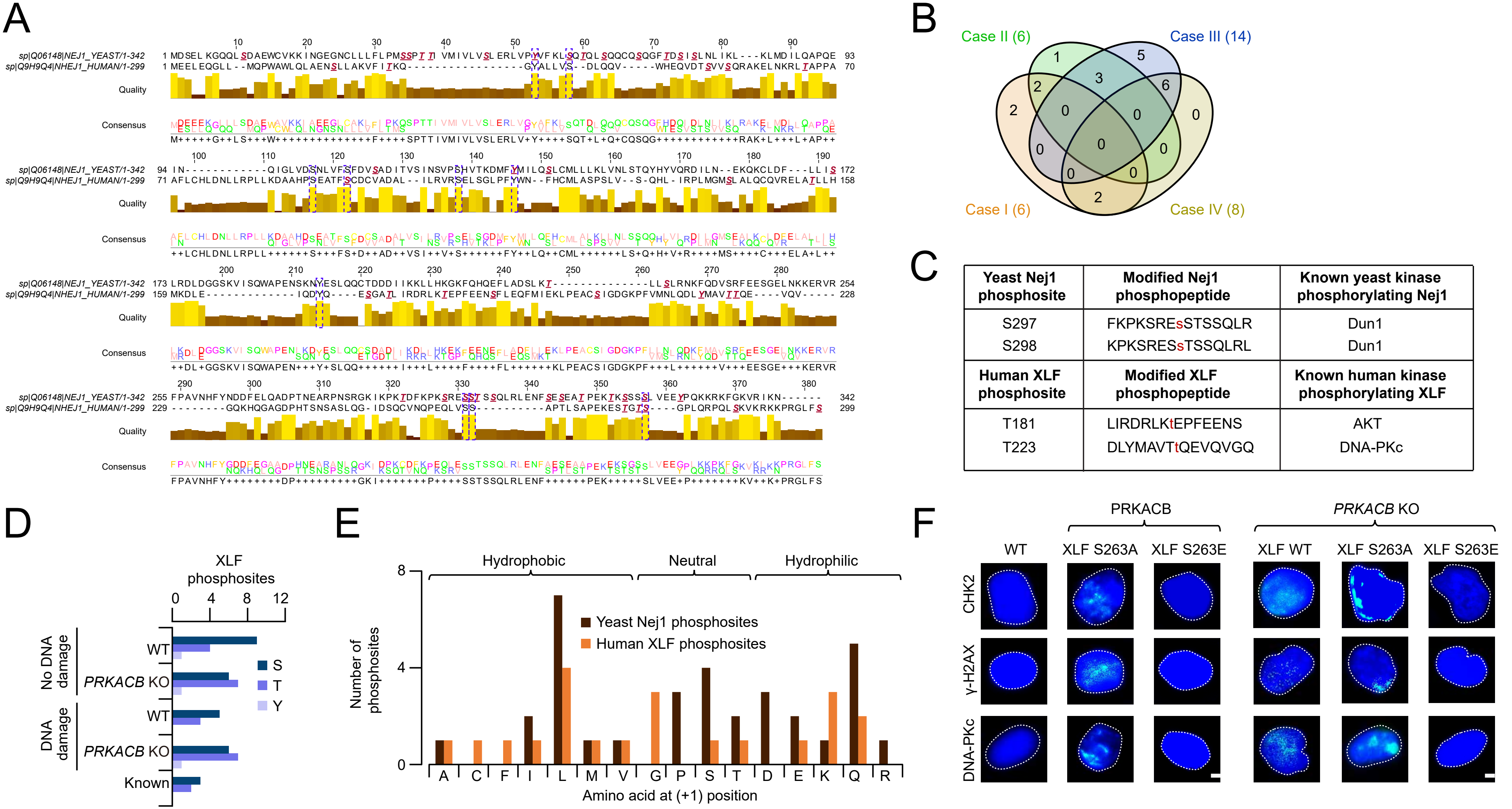

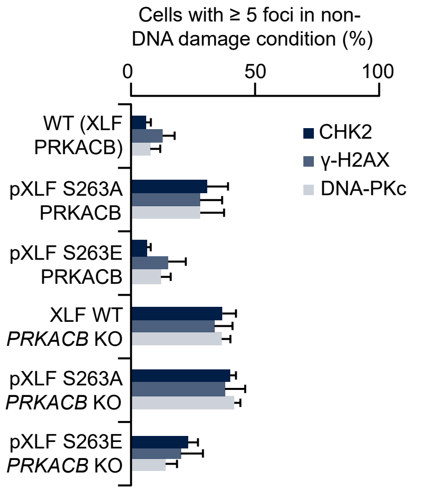

Supplementary Figure S5. Human PRKACB phosphorylation of XLF, and conserved residues and phosphosites between yeast Nej1 and human XLF (i.e. NHEJ1). (**A**) Conserved (dotted line) and phosphorylated (red letter in italics) residue of yeast Nej1 and human XLF. Non-phosphorylated residue is italicized and not emphasized in red. (**B**) Venn diagram showing human XLF phosphosite overlap in each of the PRKACB dependent or independent cases (see Supplementary Table S5 for details). (**C**) Yeast Nej1 and human XLF phosphosites (modified phosphorylated residue shown in red) regulated by known yeast or human kinases. (**D**) Number of XLF phosphosites (STY) modified in response to DNA damage and non-DNA damage in wild-type (WT) and *PRKACB* KO U20S cells. (**E**) Analysis of all detected hydrophobic, neutral, and hydrophilic phosphosites in yeast and human phosphoproteomics with various amino acids at position +1. (**F**) CHK2, γ-H2AX, and DNA-PKc foci (n = 200 cells per sample) in control sgRNA and knockout (KO) U2OS cells in non-DNA damaging condition were immunostained with anti-CHK2, γ-H2AX, and DNA-PKc antibody. Nuclei stained with DAPI are indicated with white dotted lines. Scale bar, 20 μm.

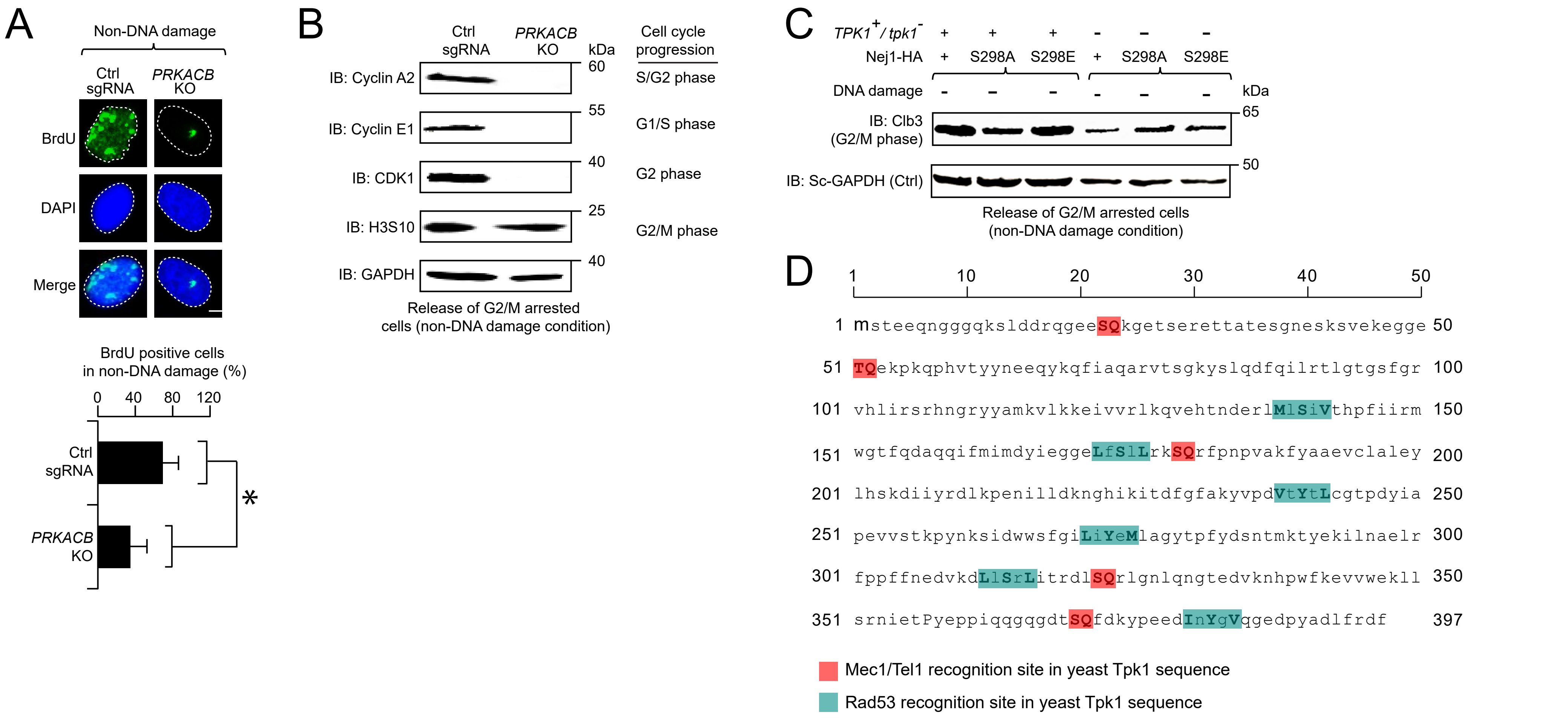

Supplementary Figure S6. Yeast *tpk1* mutant or Nej1 variants, and the human *PRKACB* knockout (KO) or XLF variants on the expression of cell cycle regulators, as well as the Tpk1 recognition sites. (**A**) Images and quantification (n = 50 cells per sample) of BrdU positive cells with anti-BrdU antibody from the immunostaining of indicated strains after a 11 h G2/M arrest, and then released into the medium with no DNA-damage. (**B,C**) Expression of cell cycle regulators in the indicated human (B) and yeast (C) strains immunoblotted (IB) by probing with protein-specific antibodies. U2OS cells containing the human strains (B) were synchronized with a double thymidine block, followed by a 11h G2/M cell cycle arrest by nocodazole, and then released into the medium with no DNA-damaging agent. In yeast JKM139-strain cultures (C), G2/M phase was arrested for 2 hrs by nocodazole treatment. (**D**) Potential Mec1/Tel or Rad53 recognition sites found in the primary sequence of yeast Tpk1.

The Conserved Tpk1 Regulates Non-Homologous End Joining Double-Strand Break Repair by Phosphorylation of Nej1, a Homolog of the Human XLF

Matthew Jessulat[1,*], Shahreen Amin[1,*], Mohsen Hooshyar[2,3*], Ramy Malty[1], Mohamed Taha Moutaoufik[1], Mara Zilocchi[1], Zoe Istace[1], Sadhna Phanse[1], Hiroyuki Aoki[1], Katayoun Omidi[2,3], Daniel Burnside[2,3], Bahram Samanfar[2,3], Khaled A. Aly[1],
Ashkan Golshani[2,3,#], Mohan Babu[1,#]

SUPPLEMENTARY METHODS

Site-directed mutagenesis in yeast and mammalian cells

Site-directed mutagenesis in yeast was carried out on a NEJ1-expressing HA-tagged plasmid using the Phusion site-directed mutagenesis kit (ThermoFisher Scientific) and phosphorylated primers containing the mutated site of interest. Mutagenized sequences were confirmed using primers listed in **Supplementary Table S1** through Sanger sequencing at Toronto's TCAG (The Centre for Applied Genomics) facility. Plasmids were inserted into *nej1*-deleted JKM139 yeast strains for chromosomal DSB repair assay, or PCR amplification of a cassette including mutagenized *nej1* containing the URA3 selection marker was transformed to create *nej1* mutations.

To create XLF (human homolog of the yeast NEJ1 (1), aka NHEJ1) non-phosphorylatable (S263A) and phosphomimetic (S263E) mutants, we used a PCR-driven overlap extension method (2). Briefly, a cDNA clone (IMAGE: 4076851; Locus: BC030986) with stop codon was gateway-cloned into pLD-puro-CnVA (containing a triple (FLAG-HIS-STREP) versatile affinity (VA) epitope tag at the N-terminus) with forward (XLF-clone-F) and reverse (XLF-clone-R) primers (**Supplementary Table S1**) to generate a wild-type XLF VA-tag. In parallel, we also designed XLF outer forward (XLF-OF) and reverse (XLF-clone-R) primers flanking the *BshT*I and *Xba*I sites, as well as inner forward and inner reverse primers corresponding to the desired mutation (XLF-S263A-IF/IR; XLF-S263E-IF/IR). The resulting amplicons were stitched using an overlap

extension PCR with XLF outer forward (XLF-OF) and reverse (XLF-clone-R) primers. After digesting the PCR product or pLD-puro-CnVA-XLF vector with *BshT*I and *Xba*I enzymes, the purified amplicons and plasmid were ligated, transformed, and colonies selected were verified using Sanger sequencing at the TCAG facility. All wild-type and mutant plasmids were deposited in Addgene and listed in the **Supplementary Table S1**.

In vitro kinase and phosphorylation-induced mobility shift assays

The kinase activity of yeast Tpk1 and Nej1 (wild-type or mutant) was determined by the luminescence signal intensity from the Kinase-Glo assay kit (Promega), following the manufacturer's protocol. Purified Nej1 and Tpk1 proteins (**Supplementary Methods**) were quantified using a bicinchoninic acid method, and 0.01 ng of Nej1 (wild-type or mutant) protein was combined with 5 µM ATP and 0.01 ng of Tpk1 recombinant protein. The luminescence signal corresponding to kinase activity was measured via Synergy multimode plate reader.

Nej1 phosphorylation by Tpk1 was assessed for changes in Nej1 electrophoretic mobility shift to DNA damage as described (3) by chromosomally integrating Nej1-HA in wild-type or *tpk1* mutant, with or without the Nej1 modifications (S298A, S298E). DNA damage in cultures was induced by the addition of 2% galactose for 1 hr. Similarly, XLF mobility shift to BLM-induced DSBs was assessed by exogenously expressing the XLF-VA (containing the FLAG) tag in wild-type or *PRKACB* knockout (KO) USOS cells, with or without the XLF modifications (S263A, S263E). In both cases, cell extracts were resolved at 12% SDS-PAGE and immunoblotted with anti-HA or anti-FLAG antibody to detect wild-type and phosphorylated Nej1 or XLF.

Sensitivity to DNA damage, clonogenic survival, and cell proliferation

Sensitivity to Homothallic switching (HO) endonuclease was conducted by logarithmically

growing yeast strains in YPD (1% yeast extract, 2% bacto-peptone, 2% glucose) or non-repressing YPEG (YP plus 2% ethanol and 3% glycerol) media at 30°C, and spotting the serially diluted cultures onto YPD or YP-galactose (2%) plates to induce DSB concurrently with the overexpression of specific gene expression on GAL-regulated plasmid vectors. The plates were incubated for 3 days at 30°C, and growth of the colonies was visually examined. U2OS cells (5x10^3 per well) transfected with control sgRNA or CRISPR gene KOs were seeded in triplicate, in a 96-well plate treated without or with bleomycin (BLM; 10 μg/ml). After 72 hrs of incubation, cells were washed with phosphate buffered saline (PBS), and the number of viable or proliferated cells was assessed following tetrazolium MTT (3-(4, 5-dimethylthiazolyl-2)-2,5-diphenyltetrazolium bromide) cell viability and proliferation assay kit, and measuring absorbance (formazan) at 570 nm using a Synergy multimode plate reader.

Phosphoproteomic screen coupled with mass spectrometry (MS)

Overnight JKM139-derived yeast strains containing Nej1-FLAG in wild-type or *tpk1* deletion background were grown in YPEG media to mid-logarithmic phase at 30°C with 200 *xg* shaking before induction of DNA damage by the addition of 2% galactose. Cells were harvested and resuspended in ice-cold extraction buffer (20 mM HEPES KOH (pH 7.4), 150 mM KOAc, 2 mM $Mg(Ac)_2$, 1 mM EGTA, 10% glycerol, 1% digitonin, 1X protease inhibitor cocktail, 1X phosphatase inhibitor cocktail II and III), followed by cell lysis via freezer mill and centrifugation for 10 min at 15,000 *x g* to separate proteins from debris. About 3 µl of anti-FLAG antibody was added to the lysate, and after 1 hr agitation at 4°C, 50 µl of µMACS protein G magnetic microbeads (Miltenyi) was added and agitated for 4 hrs at 4°C. Microbeads suspension was then passed through µMACS columns following manufacturer's instructions.

Approximately 150 μg of the digested mixture (reaction stopped by adding 1% formic acid) was desalted on disposable TopTip C-18 columns (Glygen), and lyophilized sample was subjected to titanium dioxide-metal oxide (TiO_2) phosphopeptide enrichment kit (Thermo Fisher Scientific) according to manufacturer's instructions. The samples were acidified by adding 1% trifluoroacetic acid, followed by desalting (C-18 column, Millipore) and drying in vacuum evaporator. The phosphopeptide mixture was then analyzed by an Easy-nanoflow liquid chromatography 1000 (Easy nLC; Proxeon) system coupled to an Orbitrap Elite mass spectrometer (Thermo Fisher Scientific). Detailed procedures for processing chromatographic separation, and control settings for full scanning of MS spectra acquisition are described in our earlier study (4).

Raw spectra files were analyzed with the Proteome Discoverer software ver 2.2 (Thermo Fisher Scientific) against the yeast Nej1 protein sequence FASTA file (ver. 28-Mar-2018; entry ver. 110) from the Uniprot database. Search parameters were set to 2 missed tryptic cleavages, dynamic modification on methionine oxidation, phosphorylation of STY (Ser/Thr/Tyr), one fixed modification on cysteine carbamidomethylation using precursor ion tolerances of 20 ppm, and with a fragment tolerance of 0.5 Da. Percolator was used to identify reliable phosphopeptides with a false discovery rate (FDR) of $q \leq 0.05$ to discriminate correct from decoy spectra identifications. Only the correct phosphorylation site within each phosphopeptide that was predicted by proteome discover using a phosphoRS algorithm is assigned with a site probability score.

Phosphoproteomics in mammalian cell line was carried out in U2OS wild-type or CRISPR-mediated *PRKACB* KO cells (3 x 10^6 per well) carrying the XLF (a Nej1 human homolog)-FLAG, treated with or without bleomycin (10 μg/ml), in triplicate. About 80-90% confluent cells were washed with PBS and then detached with 0.5 mM EDTA with 1x phosphatase inhibitor cocktails

I-III in PBS buffer, followed by centrifugation at 300 *x*g for 10 min. The pelleted cells were lysed in RIPA buffer (150 mM NaCl, 50 mM Tris-HCl (pH 7.5), 0.1% sodium dodecyl sulfate, 1% Na deoxycholate, 1% NP-40, 1mM EDTA), followed by 20 strokes with a pre-chilled glass Dounce homogenizer, 20 strokes up and down using 3 ml syringe with 23-gauge needle, and centrifugation at 14,000 *x*g for 20 min at 4°C. After removing the debris, the lysate was incubated with 100 µl of anti-FLAG microbeads (Miltenyi), followed by affinity purification and TiO_2 phosphopeptide enrichment (Thermo Fisher Scientific) using the aforesaid procedure as with yeast.

Raw spectra files were analyzed with MSGF+ (ver. 2017.01.13) against the human XLF protein sequence FASTA file (ver. 28-Mar-2018) from the Uniprot database in a target-decoy mode, and Percolator was used to detect phosphopeptides at $q \leq 0.05$. Search parameters were set to: (1) 2 missed tryptic cleavages, dynamic modification on methionine oxidation, (3) STY phosphorylation, (4) a fixed modification on cysteine carbamidomethylation using parent mass tolerances of 20 ppm, and (5) isotope error range of -1 to 2. Phosphosites detected in at least 2 of the 3 replicates or those present in ≥ 2 phosphopeptides or previously reported in PhosphoSite Plus (a public phosphopeptide repository) were considered for analyses.

Since we failed to detect in vivo phosphorylation of XLF S263 in U2OS cells under the aforementioned phosphoproteomics conditions, we repeated the XLF phosphorylation-site mapping screen several times under different experimental conditions. This includes: (1) growing U2OS cells in large quantities to ensure the presence of XLF protein in sufficient amounts; (2) alternating phosphopeptide enrichment method such as immobilized metal affinity chromatography as opposed to the reported TiO_2-based method; and (3) purifying the U2OS wild-type or *PRKACB* KO cell lysates with XLF antibody instead of affinity purifying the samples

carrying the XLF-FLAG with anti-FLAG microbeads. Also, we analyzed the raw mass spectra profiles using other search engines such as Sequest or MaxQuant, and relaxed the parent mass tolerance from stringent 20 ppm to the standard use of 10 ppm. Regardless of these changes, we failed to observe the phosphorylation of XLF at S263. Besides, factors such as length, hydrophobicity and charge of the protein (5) can affect the chromatographic properties and ionization efficiencies, which may have resulted in the failure to detect XLF S263 site.

Affinity purification (or immunoprecipitation) combined with MS

The C-terminal FLAG-tagged Tpk1 or Nej1 fusion strains created with a KanMX selectable marker integrated by homologous recombination in the yeast chromosome were grown, in sextuplicate, to mid-log phase in 1 L of YPEG media (to keep the growth condition consistent with rest of the experimental procedures). After the lysis of harvested cells by freezer mill, the extracts were purified using anti-FLAG magnetic microbeads, and subjected to μMACS columns (Miltenyi) following manufacturer's instructions. Sample preparation for digestion, and chromatographic separation of peptides on Easy nLC and orbitrap mass spectrometer are described in detail in our earlier publications (4,6). The MS spectra from each tagged protein was subsequently mapped to reference yeast protein sequences (downloaded from Uniprot database) using a MaxQuant (ver. 1.6.1.0) search engine for peptide identifications. Precursor mass tolerance was set to 20 ppm, while allowing partial tryptic enzyme and 2 site missed cleavages. Interacting proteins were considered as high confidence as long as they: (1) pass through an FDR cut-off of $q \leq 0.05$, (2) are not present in the untagged control strain, and (3) have been identified in at least 3 of the 6 replicates from each tagged protein purification.

In the case of PRKACB, a human homolog of Tpk1, pull down experiments were performed in triplicate, at 80% confluent U2OS cells (2 x 150 mm tissue culture dishes; 10^7 cells per dish) using

protein specific antibody, following the procedure we previously described (6). The MS spectra were mapped to the reference human protein sequences using the SEQUEST (ver.27-rev.9) search engine by setting the precursor mass tolerance to 3 Da, as well as allowing partial tryptic enzyme and 2 site missed cleavages. The STATQUEST filtering algorithm was then applied to putative SEQUEST search results to assign statistical confidence as we described (7). Spectral counts of the interacting proteins filtered at 95% confidence ($p \leq 0.05$) threshold as assigned by STATQUEST, and retaining those detected in two of the three replicate pull down experiments were considered as high quality interactions.

Purification of yeast Tpk1 and Nej1 recombinant proteins

The plasmids carrying the HA-tagged Tpk1 and Nej1 (wild type, non-phosphorylatable or phosphomimetic mutants) were grown to mid-logarithmic phase in 500 ml YP-galactose (2%) media. Harvested cells were resuspended in ice-cold extraction buffer, and frozen in liquid nitrogen, before lysis with a freezer mill. The lysed cells were clarified by centrifugation at 200 *xg* for 10 min, and to the resulting supernatant, 3 μL anti-HA (Sigma-Aldrich) antibody and 50 μL μMACS Protein G beads (Miltenyi) were added. The μMACS columns were equilibrated with 250 μL IPLB buffer (20 mM HEPES KOH (pH 7.4), 150 mM KOAc, 2 mM $Mg(Ac)_2$, 1 mM EGTA, 0.6M sorbitol, 1 x protease inhibitor cocktail), prior to loading the lysates onto columns.

After washing the columns twice with 1000 μL IPLB buffer, and once with the same buffer without protease inhibitor cocktail, 25 μL of elution buffer (2M Urea; 100mM Glycine, pH 2.8) was added to the column. After 10 min of incubation, an additional 100 μL of elution buffer was added, and the elution was collected into tubes containing 25 μL of 1 M Tris-HCl (pH 8.0). The eluted sample was then dialyzed for 48 hrs at 4°C in buffer A (0.4 M Urea, 50 mM Tris-HCl (pH 8.0), 3 mM

reduced glutathione (GSH), 0.3 mM oxidized glutathione (GSSG), 10% glycerol) using a 3K molecular weight cutoff dialysis membrane tubing (Fisher Scientific). The dialyzed proteins were cleared by centrifugation, and the supernatant fraction was stored at -80°C, until use. Protein purity was assessed by SDS-PAGE gel using Coomassie blue staining, and the protein identity was confirmed by immunoblotting using anti-HA antibody.

Immunoblotting

U2OS cells grown in 10 mm plates, washed with PBS, trypsinized, detached, and spun down. The pellet was resuspended in lysis buffer (50 mM HEPES pH 7.5, 150 mM NaCl, 10% glycerol, 1% Triton X-100, 1.5 mM $MgCl_2$, 1 mM EGTA). Samples were inverted for 25 min at 4°C after which they were pelleted, and the supernatant was used for protein detection. For detecting PRKACB and XLF interaction in U2OS cells treated with or without bleomycin, the lysate was immunoprecipitated with XLF antibody, and probed with PRKACB antibody. Samples were separated on 10% SDS-PAGE gel in Tris-glycine running buffer. After separation, proteins were transferred to nitrocellulose membrane, probed with primary and HRP-conjugated secondary antibodies, detected by chemiluminescence, and imaged using LI-COR imaging system. Also, the immunoprecipitates of yeast Dun1-HA or Nej1-HA (wild-type or mutants) tagged proteins transformed in Tpk1-TAP were visualized by anti-HA or anti-TAP antibody.

To assay potential protein expression regulation by Tpk1 and determine steady-state protein levels after DNA damage induction and across the cell cycle, JKM139-strain cultures were grown overnight in YPEG media before cell cycle arrest by the addition of nocodazole (20μg/ml) and incubation for 2 hrs. DNA damage was induced by the addition of 2% galactose and incubation continued for an additional 30 min, before release by washing cells three times in fresh YPD media (removing the nocodazole and replacing galactose with glucose to end DNA damage induction).

5ml of cell culture was collected at set time points and immediately disrupted via centrifugation, resuspension in 0.5ml IPLB with protease inhibitor cocktail, and vortexing with glass beads for 5 min. Cell lysate was collected as supernatant after centrifugation and was frozen immediately.

Generation of stable CRISPR gene KOs

Oligonucleotides encoding non-targeting control and PRKACB or XRCC5 selective sgRNAs targeting the gene sequence, designed using CRISPR online tool, were phosphorylated, annealed and cloned in LentiCRISPRv2-blasticidin plasmid as described before (8). Likewise, XLF sgRNA was cloned into the LentiCRISPRv2-puromycin plasmid. Ligated product was transformed in Stbl3 competent cells, and ampicillin positive single colonies were selected and grown in Luria-Bertani liquid media for plasmid extraction. Correct insertion of sgRNAs was confirmed by Sanger DNA sequencing at Toronto's TCAG facility.

About 2.5 µg of sequence-verified LentiCRISPRv2-blasticidin or puromycin plasmid DNA containing non-targeting control, and PRKACB, XRCC5 or XLF sgRNAs were combined with lentiviral packaging plasmids (psPAX2, pMD2.G) and transduced in HEK293T cells. After 48 and 72 hrs of transfection, supernatant containing the lentivirus removed from HEK293T cultures was combined, and filtered through 0.4 µm low-protein binding syringe filters to remove cell debris. The resulting lentiviral supernatant was then added to U2OS cells (10^6 cells per 6 cm dish) for 24 hrs after which cells that stably express LentiCRISPRv2-blasticidin or puromycin expression cassette were selected using blasticidin (5 µg/ml) or puromycin (2.5 µg/ml) for 14 days. In case of double KOs, U2OS cells were stably expressed with both LentiCRISPRv2-blasticidin and puromycin, following the aforesaid procedure. After the selection was complete, cells were verified for successful gene KO compared to non-targeting sgRNA expressing cells by immunoblotting using protein-specific antibody.

Immunofluorescence staining for CHK2, γ-H2AX, and DNA-PKc foci

Both non-targeting control sgRNA and stable CRISPR gene KO U2OS cells (5×10^4 cells per well) seeded onto coverslips in 6-well plates in Dulbecco's modified Eagle's media (supplemented with 10% fetal bovine serum, 1% penicillin-streptomycin mix at 37°C, 5% CO_2) containing blasticidin or puromycin. After 48 hrs of incubation, the media is refreshed and cells were fixed in 4% paraformaldehyde in PBS for 15 min, permeabilized for 10 min with PBS containing 0.3% Triton X-100 and blocked with PBS containing 1% BSA/2% glycine/0.1% Tween 20 for 1 hr.

The cells were immunostained with CHK2, **γ**-H2AX, or DNA-PKc (Abcam) primary antibody (diluted in PBS encompassing 1% BSA, 2% glycine, 0.1% Tween 20) overnight at 4°C. After washing the cells 3 times (5 min per wash) with PBS containing 0.1% Tween 20, cells were incubated with secondary Alexa Fluor® 488 conjugated antibody in PBS containing 1% BSA, 2% glycine, and 0.1% Tween 20 for 1 hr at room temperature. The cells were once again washed with PBS containing 0.1% Tween 20, and the DNA was counterstained by incubating the cells with Fluoroshield histology mounting media containing 4′,6-diamidino-2-phenylindole (DAPI, Sigma-Aldrich). Images were acquired in a Zeiss fluorescent microscope with 20X objective, and quantification was done by counting positive cells (n = 200 nuclei per sample) using ImageJ or automatic acquisition of images using Zeiss Zen microscope software.

Alkaline comet assay

U2OS cells (2×10^3 cells per slide) carrying non-targeting control sgRNA and CRISPR gene KOs, treated with or without bleomycin for 3 hrs was resuspended in 0.8% low-melting-temperature agarose (Sigma-Aldrich), and spread on 1% low-melting-temperature agarose precoated slide. After solidification, the slides were immersed in chill lysis buffer (2.5 M NaCl, 0.1 M EDTA, 10 mM Tris-HCl (pH 10.0), 10% DMSO, 1% Triton X-100) for 1 hr. Slides were washed for 5 min

in the neutralization buffer (0.4 M Tris, pH 7.5) at 4°C, and immersed in chilled alkaline solution (300 mM NaOH, pH 13.0; 1 mM EDTA) for 30 min to unwind supercoiled DNA. Electrophoresis was then performed with the same alkaline electrophoresis solution (300 mM NaOH, pH 13.0; 1 mM EDTA) at 4°C with an electrophoretic run set at 25V and 300 mA for 25 min. The slides were then washed three times with the neutralization buffer for 10 min. Prior to visualization, the slides were stained with SYBR Gold (1:10,000 Tris-borate-EDTA buffer; Thermo Fisher Scientific) for 30 min in the dark, fixed with ice-cold ethanol, and dried for 30 min. Comets were viewed in the Zeiss Observer Z1 inverted microscope with Colibri 2 epifluorescence on the Alexa Fluor 488 channel. From each slide, 250 comets were imaged randomly with a Zeiss microscope, and scored for DNA in comet Tail, comet tail length, and comet tail moment using comet score software.

Isolation of cytoplasmic and nuclear fractions

Cytoplasmic and nuclear extracts were isolated using chemical fractionation with protease inhibitors cocktail (PIC, Roche) as previously described (9). Briefly, cells (3×10^6) washed in cold buffer A (10 mM HEPES, 10 mM KCl, 1.5 mM $MgCl_2$, 1X PIC) was resuspended in 70 µl of working buffer A with 0.1% NP-40, followed by centrifugation at 20,000 *x g* for 10 min. The supernatant, encompassing cytoplasmic proteins, was collected for further use. The resulting pellet was washed in cold buffer A by resuspending and centrifugation at 20,000 *x* g. The supernatant is discarded, and the pellet is resuspended in 50 µl of working buffer B (20 mM HEPES, 420 mM NaCl, 1.5 mM $MgCl_2$, 0.2 mM EDTA, 25% glycerol, 1X PIC), and centrifuged at 20,000 × *g* for 15 min. The supernatant, consisting nuclear proteins was stored at -80°C in equal volume of buffer C (20 mM HEPES, 50 mM KCl, 0.2 mM EDTA, 20% glycerol, 1X PIC) for immunoblotting.

BrdU incorporation using immunofluorescence and immunocytochemistry

Cell cycle synchronization protocol was adapted from previous work (10), while BrdU

incorporation (Abcam) was conducted as per manufacturer's instruction. In brief, for immunofluorescence, U2OS cells (2.5×10^4 /well) were grown in a 96-well plate containing 100 µl of DMEM medium, and synchronized at G1/S phase with 2 mM double thymidine block for 20 hrs at 37°C with 5% CO_2. Cells were then washed twice with pre-warmed PBS, and 100 µL of DMEM is added to each well. After 5 hrs of incubation at 37°C, cells were arrested at G2/M phase by nocodazole treatment (50 ng/mL) for 11 hrs, and then released into the DMEM medium containing bleomycin for 3 hrs. Cells washed with 1X PBS twice were incubated with BrdU (10 µM) overnight, followed by washing thrice with PBST to remove residual BrdU. Fixed cells with 100% methanol for 15 min were permeabilized with 0.1% Triton X-100, and blocked with 3% fetal bovine serum in PBST for 1 hr. Anti-BrdU primary antibody subjected cells were then incubated overnight prior to washing with PBST and further incubation with secondary Alexa Fluor® 488 conjugated antibody. Cells washed for 5 times with PBST was subjected to 50µl of DAPI (as internal control for normalization), and then fluorescence was measured using Synergy multimode plate reader at 450/530 nm (excitation/emission wavelength) for BrdU incorporation, and 350/430 nm for DAPI. Following the aforesaid method, BrdU incorporation for immunocytochemistry was performed in a 6-well plate, and images acquired in a Zeiss fluorescent microscope were quantified for positive BrdU cells (n = 50 per sample) via manual inspection.

REFERENCES

1. Callebaut, I., Malivert, L., Fischer, A., Mornon, J.P., Revy, P. and de Villartay, J.P. (2006) Cernunnos interacts with the XRCC4 x DNA-ligase IV complex and is homologous to the yeast nonhomologous end-joining factor Nej1. *J Biol Chem*, **281**, 13857-13860.
2. Heckman, K.L. and Pease, L.R. (2007) Gene splicing and mutagenesis by PCR-driven overlap extension. *Nat Protoc*, **2**, 924-932.
3. Ahnesorg, P. and Jackson, S.P. (2007) The non-homologous end-joining protein Nej1p is a target of the DNA damage checkpoint. *DNA Repair (Amst)*, **6**, 190-201.
4. Jessulat, M., Malty, R.H., Nguyen-Tran, D.H., Deineko, V., Aoki, H., Vlasblom, J., Omidi, K., Jin, K., Minic, Z., Hooshyar, M. *et al.* (2015) Spindle Checkpoint Factors Bub1 and Bub2 Promote DNA Double-Strand Break Repair by Nonhomologous End Joining. *Mol Cell Biol*,

35, 2448-2463.
5. Dephoure, N., Gould, K.L., Gygi, S.P. and Kellogg, D.R. (2013) Mapping and analysis of phosphorylation sites: a quick guide for cell biologists. *Mol Biol Cell*, **24**, 535-542.
6. Malty, R.H., Aoki, H., Kumar, A., Phanse, S., Amin, S., Zhang, Q., Minic, Z., Goebels, F., Musso, G., Wu, Z. *et al.* (2017) A Map of Human Mitochondrial Protein Interactions Linked to Neurodegeneration Reveals New Mechanisms of Redox Homeostasis and NF-kappaB Signaling. *Cell Syst*, **5**, 1-14.
7. Babu, M., Vlasblom, J., Pu, S., Guo, X., Graham, C., Bean, B.D., Burston, H.E., Vizeacoumar, F.J., Snider, J., Phanse, S. *et al.* (2012) Interaction landscape of membrane-protein complexes in Saccharomyces cerevisiae. *Nature*, **489**, 585-589.
8. Shalem, O., Sanjana, N.E., Hartenian, E., Shi, X., Scott, D.A., Mikkelsen, T.S., Heckl, D., Ebert, B.L., Root, D.E., Doench, J.G. *et al.* (2014) Genome-scale CRISPR-Cas9 knockout screening in human cells. *Science*, **343**, 84-87.
9. Amin, S., Kumar, A., Nilchi, L., Wright, K. and Kozlowski, M. (2011) Breast cancer cells proliferation is regulated by tyrosine phosphatase SHP1 through c-jun N-terminal kinase and cooperative induction of RFX-1 and AP-4 transcription factors. *Mol Cancer Res*, **9**, 1112-1125.
10. Harper, J.V. (2005) Synchronization of cell populations in G1/S and G2/M phases of the cell cycle. *Methods Mol Biol*, **296**, 157-166.

Supplementary Table Legends

Supplementary Table S1. List of strains, plasmids, primers, and antibodies used in this study.

Supplementary Table S2. PKA catalytic subunits, HR, NHEJ, and MRX genes share synergistic interactions with DNA damage-related factors in untreated and MMS-specific conditions.

Supplementary Table S3. DNA repair-related proteins physically associated with Tpk1 and Nej1 chromosomally FLAG-tagged strains.

Supplementary Table S4. Tpk1 (Sheet 1) or PRKACB (Sheet 2) dependent or independent Nej1 or XLF phosphorylation sites in the presence or absence of DNA damage.

Supplementary Table S5 PRKACB interacting proteins involved in DNA repair-related processes.
